# Immune tolerance against infused FVIII in hemophilia A is mediated by PD-L1^+^ Tregs

**DOI:** 10.1172/JCI159925

**Published:** 2022-11-15

**Authors:** Janine Becker-Gotot, Mirjam Meissner, Vadim Kotov, Blanca Jurado-Mestre, Andrea Maione, Andreas Pannek, Thilo Albert, Chrystel Flores, Frank A. Schildberg, Paul A. Gleeson, Birgit M. Reipert, Johannes Oldenburg, Christian Kurts

**Affiliations:** 1 Institute of Molecular Medicine and Experimental Immunology (IMMEI), Rheinische Friedrich-Wilhelms-Universität, Venusberg Campus 1, Bonn, Germany.; 2 Department of Biochemistry and Pharmacology, Bio21 Molecular Science and Biotechnology Institute, The University of Melbourne, Parkville, Victoria, Australia.; 3 Institute for Experimental Hematology and Transfusion Medicine (IHT), Rheinische Friedrich-Wilhelms-Universität, Venusberg Campus 1, Bonn, Germany.; 4 Clinic for Orthopedics and Trauma Surgery, University Hospital Bonn, Bonn, Germany.; 5 IMC University of Applied Sciences Krems, Krems, Austria.

**Keywords:** Hematology, Immunology, Cellular immune response, Coagulation, Immunotherapy

## Abstract

A major complication of hemophilia A therapy is the development of alloantibodies (inhibitors) that neutralize intravenously administered coagulation factor VIII (FVIII). Immune tolerance induction therapy (ITI) by repetitive FVIII injection can eradicate inhibitors, and thereby reduce morbidity and treatment costs. However, ITI success is difficult to predict and the underlying immunological mechanisms are unknown. Here, we demonstrated that immune tolerance against FVIII under nonhemophilic conditions was maintained by programmed death (PD) ligand 1–expressing (PD-L1–expressing) regulatory T cells (Tregs) that ligated PD-1 on FVIII-specific B cells, causing them to undergo apoptosis. FVIII-deficient mice injected with FVIII lacked such Tregs and developed inhibitors. Using an ITI mouse model, we found that repetitive FVIII injection induced FVIII-specific PD-L1^+^ Tregs and reengaged removal of inhibitor-forming B cells. We also demonstrated the existence of FVIII-specific Tregs in humans and showed that such Tregs upregulated PD-L1 in patients with hemophilia after successful ITI. Simultaneously, FVIII-specific B cells upregulated PD-1 and became killable by Tregs. In summary, we showed that PD-1–mediated B cell tolerance against FVIII operated in healthy individuals and in patients with hemophilia A without inhibitors, and that ITI reengaged this mechanism. These findings may impact monitoring of ITI success and treatment of patients with hemophilia A.

## Graphical abstract

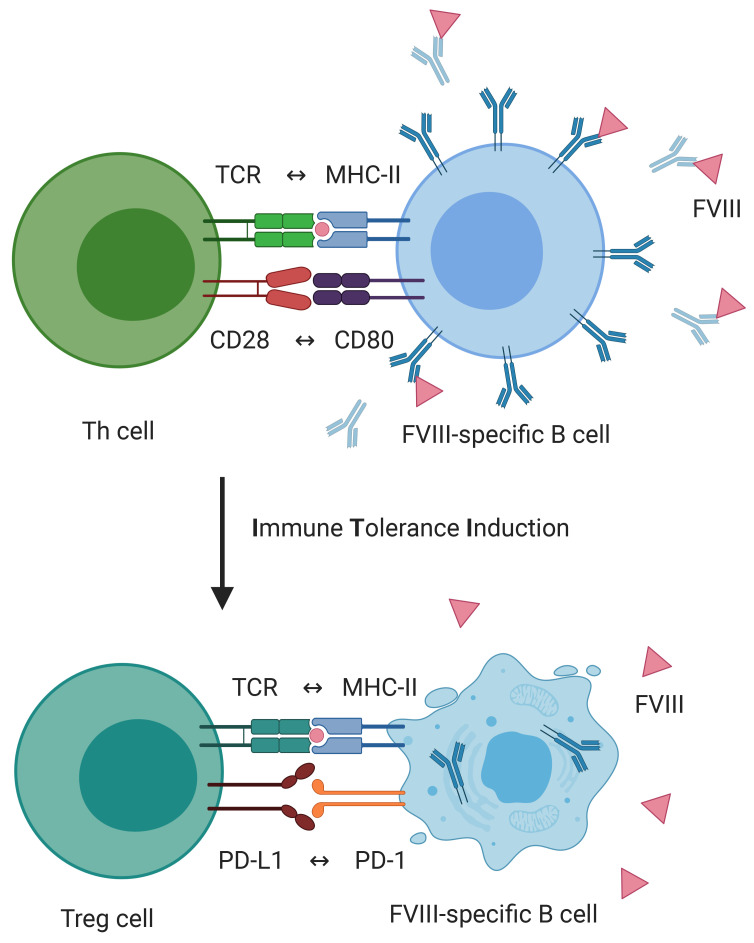

## Introduction

Hemophilia A is an X chromosome–linked inherited bleeding disorder, with an incidence of 1 in 5,000 male births (1). It results from mutations of the *F8* gene coding for coagulation factor VIII (FVIII), reducing its plasma levels or activity (2). Patients with hemophilia A suffer from lifelong spontaneous or traumatic bleeding tendencies (3). Most patients receive protein replacement therapy with plasma-derived or recombinant FVIII administered i.v. The main therapeutic complication is the formation of inhibitory anti-FVIII antibodies, which develop in approximately 30% of the hemophilia A patients (4–6). Such inhibitors neutralize the infused FVIII protein, leading to increased hemophilia A morbidity and mortality (7). The type of *F8* mutation is a major risk factor, but even among patients completely lacking FVIII secretion (about 80% of all cases), the risk of inhibitors varies between 20% and 80%. Other risk factors include the FVIII dose at first treatment and environmental factors. At present, it is not possible to predict which patients will develop inhibitors (8, 9).

Treatment of hemophilia A patients with inhibitors is very expensive and represents a great burden to the patients (10). A bispecific antibody that mimics the function of FVIII has been approved for hemophilia A patients with and without inhibitors (11, 12). However, the mainstay approach to induce FVIII-specific tolerance is the so-called immune tolerance induction therapy (ITI) (13, 14). ITI involves repetitive injections of high FVIII doses over 1 to 3 years (15). This approach is reminiscent of an immunological phenomenon described almost 50 years ago termed “high-zone tolerance,” which denotes the empiric observation that repetitive application of large antigen doses often induces immune tolerance (16, 17). A widely used, successful protocol for ITI is the Bonn Protocol, which has been entirely developed on an empirical basis (14, 18). However, approximately 20% to 40% of patients undergoing ITI do not achieve long-lasting peripheral tolerance (19, 20). The mechanisms underlying ITI are incompletely understood, hampering the monitoring and optimization of therapy for hemophilia patients.

Forty years ago, high-zone immune tolerance had been suggested to be mediated by suppressor T cells (21). The existence of suppressor T cells has long remained controversial and was not accepted until regulatory T cells (Tregs) were characterized more than 15 years later (22). More recent studies demonstrated that transferred or in vivo–induced Tregs can suppress inhibitor formation in mice (23–25), but the underlying mechanisms remained largely unclear. Tregs are widely known as suppressors of autoreactive T cells (26–28), but they can also suppress autoantibody (auto-Ab) production (29, 30). We previously proposed that they do so by directly suppressing autoreactive B cells in an antigen-specific and contact-dependent manner via the programmed cell death protein 1 (PD-1, CD279) signaling pathway (31, 32). PD-1 is an activation-induced member of the extended CD28/CTLA4 family, best known in the context of suppressing conventional T cell responses (33–35). PD-1 has been associated with T cell exhaustion in chronic viral infections (36, 37) and allows tumors to escape cytotoxic T lymphocyte (CTL) surveillance (38). PD-1–blocking antibodies have emerged as powerful anticancer drugs (39). However, patients treated with such PD-1–blocking antibodies often develop auto-Abs (40). Auto-Abs occur also in PD-1–knockout mice (41). The exact role of PD-1 in the formation of alloantibodies is unclear.

Experimental research on hemophilia and FVIII inhibitors has relied on HemA mice in which FVIII is nonfunctional (42–44). Injection of FVIII into these mice causes the formation of inhibitor-forming B cells and circulating inhibitors (45–47). However, these inhibitors persisted over many months, precluding their use as experimental readouts for ITI studies. This problem has hindered experimental progress in inhibitor research. Here, we report a method to detect and characterize FVIII-specific B cells and showed that these were tolerized by PD-1 in FVIII-competent individuals and after successful ITI. This knowledge allowed designing an assay to monitor ITI success in hemophilia A patients.

## Results

### PD-1 expression on FVIII-specific B cells is diminished in HemA mice.

**Figure 1:**
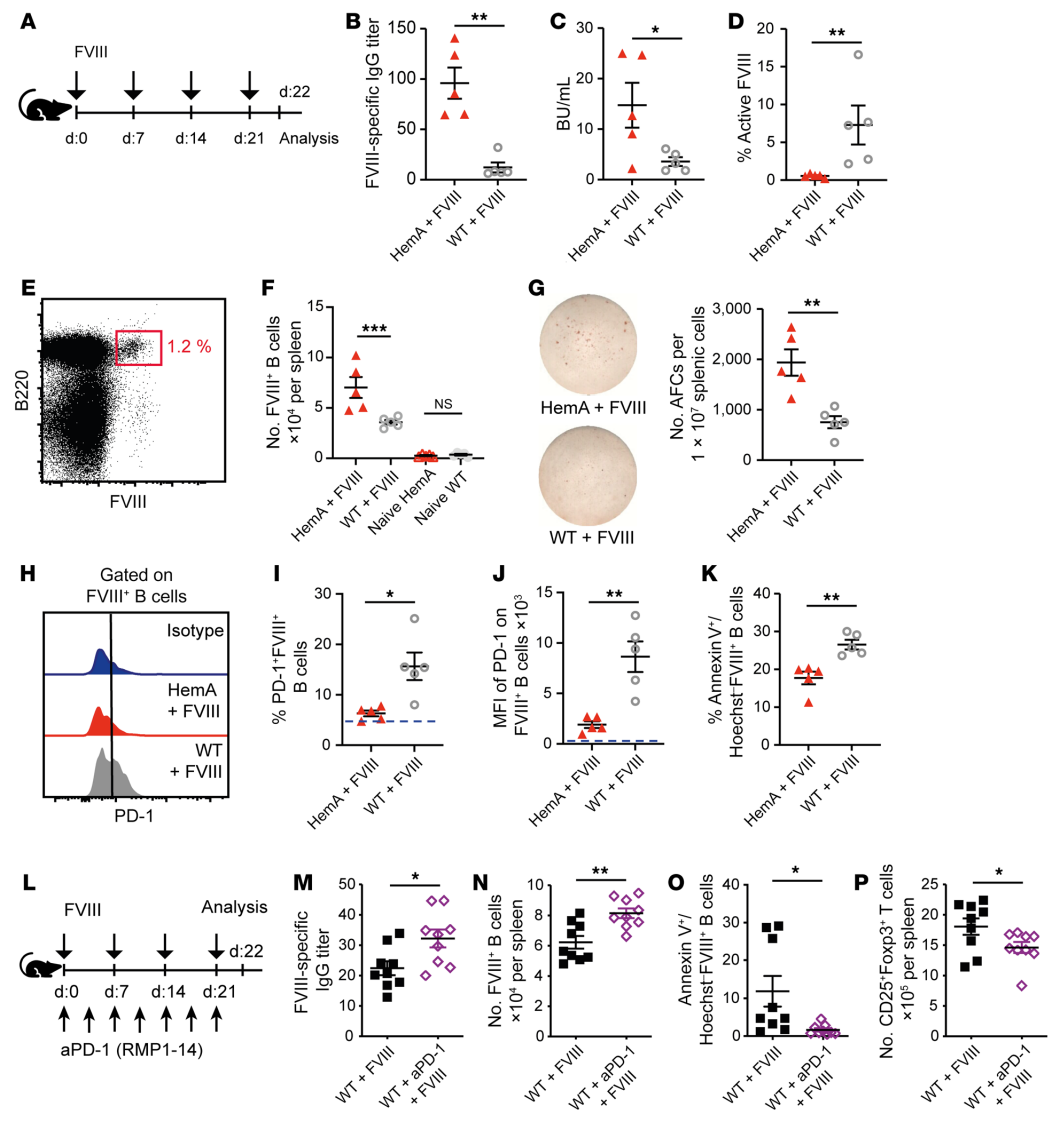
PD-1 suppresses the formation of FVIII-inhibiting antibodies in vivo. (**A**) Experimental scheme for **B**–**K**. (**B** and **C**) ELISA quantification of FVIII-specific IgG antibody titers (**B**) and Bethesda units (BU) (**C**) in the serum of HemA (red triangles) and WT mice (white circles) 22 days after weekly injections of 2 IU recombinant human FVIII (rhFVIII). (**D**) Percentage of active FVIII in the plasma of HemA or WT mice. (**E**) Gating strategy for splenic FVIII-specific B cells of mice treated once a week with rhFVIII. (**F**) Quantification FVIII-specific B cell numbers in spleens of naive HemA (*n* = 5) and WT mice (*n* = 5) or after rhFVIII treatment by flow cytometry. (**G**) Left: Representative ELISpot analysis, after coating with rhFVIII and 4-hour incubation with splenocytes. Right: Number of antibody-forming cells (AFCs) per 10^7^ splenocytes. (**H**) Representative histograms of PD-1 expression on FVIII^+^ B cells. (**I**) Proportion of PD-1–expressing FVIII^+^ B cells; the blue dashed line represents the PD-1 expression of naive B cells. (**J**) PD-1 expression by FVIII^+^ B cells. (**K**) Early apoptotic cells presented as percentage of annexin V^+^Hoechst^–^ FVIII-specific B cells after in vitro restimulation with 0.25 μg rhFVIII overnight. (**L**) Experimental setting for **M**–**P**. (**M** and **N**) ELISA-based quantification of FVIII-specific IgG antibody titers in the serum (**M**) and numbers of FVIII-specific B cells in spleens (**N**) of WT mice weekly injected with 2 IU/mouse rhFVIII and treated with an anti–PD-1 antibody (aPD-1, purple) or not (black). (**O**) Early apoptotic cells presented as percentage of annexin V^+^ and Hoechst^–^ FVIII-specific B cells after in vitro restimulation with 0.25 μg rhFVIII overnight. (**P**) Number of CD4^+^Foxp3^+^ Tregs in the spleen of treated mice. *P < 0.05; **P < 0.01; ***P < 0.001 by unpaired 2-tailed Student’s *t* test (**B**–**P**) or 1-way ANOVA with Bonferroni’s post hoc test (**F**). NS, not significant.

To clarify the mechanisms underlying the formation of neutralizing antibodies against transfused FVIII in hemophilia A, we used mice lacking functional FVIII protein (HemA mice) as a model (42). As HemA mice were maintained on the C57BL/6 × 129S F1 genetic background, we used C57BL/6 × 129S F1 mice as wild-type (WT) controls. These mice were injected 4 times in weekly intervals with recombinant human FVIII (experimental scheme in Figure 1A). After 22 days, robust anti-FVIII titers as well as inhibitors were detected in HemA mice injected with FVIII, but not in WT control mice (Figure 1, B and C). In line with enhanced inhibitor titers, significantly less active FVIII was detected in HemA mice compared with the WT control (Figure 1D). This indicated immune tolerance against FVIII in WT, and not in HemA mice, mimicking the situation in hemophilia A patients. To determine whether FVIII tolerance operated on the B cell level, we designed a flow cytometric staining protocol to identify FVIII-specific B cells (Figure 1E and Supplemental Figure 1, A–C; supplemental material available online with this article; https://doi.org/10.1172/JCI159925DS1). This revealed higher numbers of such B cells in HemA mice injected with FVIII compared with injected WT mice, whereas the size of the naive compartment was comparable in both groups (Figure 1F). The presence of FVIII-specific B cells in WT mice suggests defective deletion, receptor editing, or suppression of FVIII-specific B cells in HemA mice. This result was confirmed by ELISpot analysis, which showed increased numbers of antibody-secreting (plasma) cells in HemA mice (Figure 1G).

The technical ability to identify FVIII-specific B cells enabled us to study the molecular mechanism underlying FVIII tolerance. To this end, we analyzed such B cells for the expression of known inhibitory surface proteins. Notably, the percentage of FVIII-specific B cells expressing the immune checkpoint inhibitor PD-1 and the level of PD-1 expression on these B cells were lower in HemA mice than in WT mice (Figure 1, H–J). PD-1 expression on non–FVIII-specific B cells was unchanged (Supplemental Figure 2, A–C). Another inhibitory receptor, Fas (CD95), was unchanged on FVIII-specific B cells (Supplemental Figure 2, D and E). In contrast, the costimulatory molecule CD80 was significantly upregulated on FVIII-specific B cells in HemA mice treated with FVIII (Supplemental Figure 2, F and G). Interestingly, only the proportion of PD-1^+^ FVIII-specific B cells was lower in HemA mice than in WT controls, but not FVIII-specific B cells additionally expressing CD80 or Fas (Supplemental Figure 2H). Furthermore, the enhanced PD-1 expression was associated with increased apoptosis markers on FVIII-specific B cells in WT mice compared with HemA mice (Figure 1K). This suggested that inhibitory signaling through PD-1 might prevent FVIII-specific B cells in WT mice from producing FVIII inhibitors and that this mechanism may be defective in B cells from HemA mice.

### PD-1, but not CTLA4, suppresses the formation of FVIII-inhibiting antibodies in vivo.

To investigate this hypothesis, we treated FVIII-injected WT mice twice a week with an antibody (RMP1-14) that blocks PD-1 signaling (Figure 1L). This increased FVIII-specific antibody titers as well as absolute numbers of FVIII-specific B cells in WT mice (Figure 1, M and N). Total numbers of B and T cells were not altered by RMP1-14 antibody (data not shown). As PD-1 is known to inhibit B cell survival (41), we analyzed apoptosis of FVIII-specific B cells. Indeed, more of them were apoptotic in WT mice, unless treated with PD-1–blocking antibodies (Figure 1O), indicating that PD-1 established deletional B cell tolerance toward FVIII.

These findings did not exclude the possibility that other checkpoint molecules might also be involved, especially CTLA4, which can extract costimulatory molecules from antigen-presenting cells (48, 49). In support of this notion, CD80 expression was lower on FVIII-specific B cells in WT mice than in HemA mice after injection with FVIII (Supplemental Figure 2, F and G). We therefore tested for a contribution of CTLA4 to tolerance induction by injecting CTLA4-inhibiting antibodies into WT mice (experimental scheme in Supplemental Figure 3A). However, this did not significantly alter the numbers of FVIII-specific B cells, their PD-1 and annexin V expression (Supplemental Figure 3, B–E), the FVIII inhibitor titers, or the amount of circulating and active FVIII (Supplemental Figure 3, F–H). These findings argued against a contribution of CTLA4 to the suppression of FVIII-specific B cells.

### PD-L1^+^ Tregs are necessary and sufficient to suppress FVIII-specific B cells in vivo.

Next, we asked which cell type had tolerized the B cells through PD-1. We suspected that Tregs might be involved, based on our previous study on their role in maintaining tolerance of kidney-autoantigen-specific B cells (31) and recent studies showing a reduction in inhibitor generation upon Treg transfer (23–25). In support of this notion, we noted that blocking PD-1 reduced Treg numbers in WT mice injected with FVIII (Figure 1P). To visualize PD-L1–expressing Tregs, we immunized HemA and WT mice and analyzed their splenocytes by flow cytometry following the experimental protocol shown in Figure 1A. Indeed, we detected Tregs expressing PD-L1. Both the proportions of Tregs expressing PD-L1 and PD-L1 expression were increased in WT mice (Supplemental Figure 4, A–C). By contrast, PD-L1 expression was unaltered on other T cells (T helper [Th], follicular Th [fTh], CTL, γδ T, and NKT cells; Supplemental Figure 4, D–R), arguing against a relevant role of these cells in suppressing FVIII-specific B cells.

**Figure 2:**
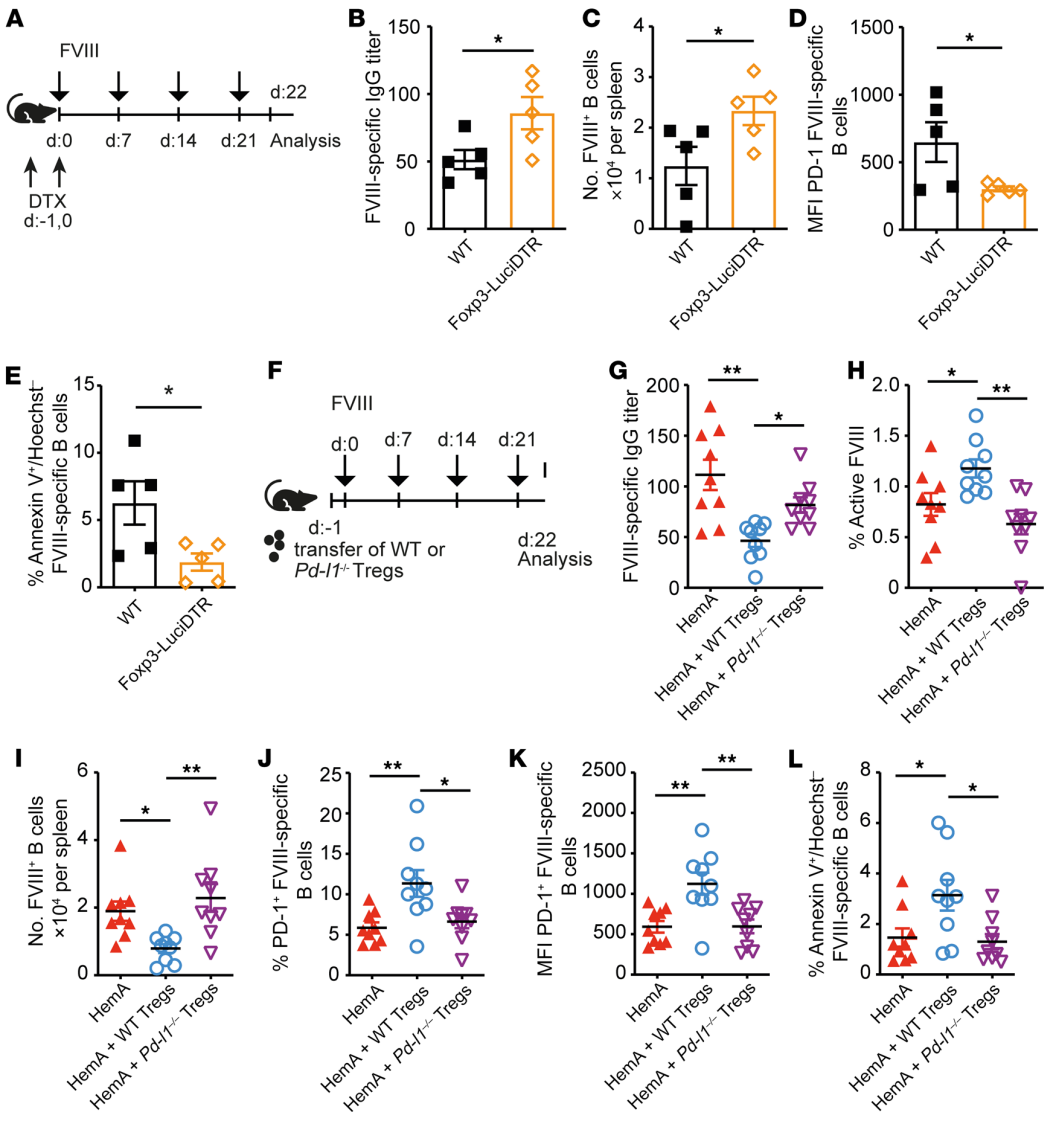
PD-L1^+^ Tregs are necessary and sufficient to suppress FVIII-specific B cells in vivo. (**A**) Experimental scheme. WT (black, *n* = 5) and Foxp3-LuciDTR (orange, *n* = 5) mice were intravenously injected with 2 IU/mouse of rhFVIII at weekly intervals. Foxp3^+^ Tregs were depleted by injecting 15 ng/g mouse DTX intraperitoneally on day –1 and 0. (**B**) ELISA-based quantification of the FVIII-specific IgG antibody titer in the serum of WT and Foxp3-LuciDTR mice. (**C**) Number of FVIII-specific B cells in spleens of WT and Foxp3-LuciDTR mice after treatment with rhFVIII by flow cytometry. (**D**) Mean fluorescence intensity (MFI) of PD-1 on FVIII-specific B cells of splenocyte suspensions. (**E**) Early apoptotic cells are presented as percentage of annexin V^+^ and Hoechst^–^ FVIII-specific B cells after in vitro restimulation with 0.25 μg rhFVIII overnight. (**F**) Experimental setup. Tregs (1 × 10^6^) isolated either from WT or *Pd-l1^–/–^* mice were injected into HemA mice. Starting on the next day, HemA (red, *n* = 9) and HemA mice that received Tregs from WT (blue, *n* = 9) or *Pd-l1^–/–^* (purple, *n* = 9) mice were intravenously injected with 2 IU/mouse of rhFVIII at weekly intervals. (**G**) FVIII-specific IgG antibody titers measured by ELISA in the serum. (**H**) The percentage of residual active FVIII in the plasma of rhFVIII-treated mice. (**I**) Number of FVIII-specific B cells in spleens of HemA mice with or without Treg transfer after rhFVIII treatment. (**J**) Proportion of splenic PD-1^+^ FVIII-specific B cells and (**K**) PD-1 MFI on FVIII-specific B cells. (**L**) Early apoptotic cells presented as annexin V^+^ and Hoechst^–^ FVIII-specific B cells after in vitro restimulation with 0.25 μg rhFVIII overnight. *P < 0.05; **P < 0.01 by unpaired 2-tailed Student’s *t* test (**B**–**E**) or 1-way ANOVA with Bonferroni’s post hoc test (**G**–**L**).

To determine whether Tregs were necessary for FVIII tolerance induction, we depleted these cells using Foxp3-LuciDTR mice on a C57BL/6 background (50) by injecting diphtheria toxin (DTX) on 2 consecutive days preceding the first FVIII injection (experimental scheme in Figure 2A). This reduced Treg numbers by more than 95% after 3 days (Supplemental Figure 5). Indeed, Treg depletion enhanced FVIII-specific antibody titers and FVIII-specific B cell numbers compared with WT controls (Figure 2, B and C). Furthermore, Treg depletion decreased PD-1 expression (Figure 2D) and apoptosis induction (Figure 2E) in FVIII-specific B cells. These findings indicated that Tregs were necessary for suppressing FVIII-specific B cells.

To investigate whether these PD-L1^+^ Tregs were also sufficient for the suppression of FVIII-specific B cells, we transferred CD4^+^CD25^+^ *Pd-l1*–competent and *Pd-l1*–deficient Tregs, which we had isolated from *Pd-l1*–deficient mice, into HemA mice 1 day before the first FVIII administration (experimental scheme in Figure 2F). Before conducting this experiment, we had verified that Treg numbers and PD-L1 expression levels (Supplemental Figure 6, A–F) and that numbers and PD-1 expression levels on FVIII-specific B cells were similar in naive HemA and WT mice (Supplemental Figure 6, G–I). *Pd-l1–*competent Tregs significantly reduced FVIII-specific antibody as well as inhibitor titers in HemA recipient mice (Figure 2G and Supplemental Figure 7) and consequently increased the amount of active FVIII, whereas *Pd-l1*–deficient Tregs were not able to do so (Figure 2H). Furthermore, the transfer of *Pd-l1*–competent Tregs decreased the numbers of FVIII-specific B cells in HemA mice and increased PD-1 expression by these B cells, as opposed to *Pd-l1*–deficient Tregs (Figure 2, I–K), consistent with a tendency toward more FVIII-specific B cell apoptosis after transfer of *Pd-l1*–competent Tregs (Figure 2L). In line with these observations, WT Tregs were able to suppress antibody-secreting B cells in vitro in a PD-1–dependent manner (Supplemental Figure 8, A and B) and the Treg-mediated suppression was antigen specific (Supplemental Figure 8, C and D). These findings showed that PD-L1^+^ Tregs were sufficient for suppressing FVIII-specific B cells.

### PD-1–stimulating agonists bypass the need for PD-L1^+^ Tregs for tolerizing FVIII-specific B cells in HemA mice.

**Figure 3:**
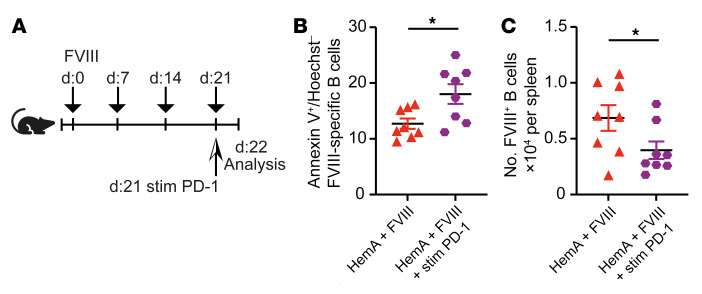
PD-1–stimulating antibodies bypass the need for PD-L1^+^ Tregs for tolerizing FVIII-specific B cells in HemA mice. (**A**) Experimental setup. HemA (red *n* = 8, purple *n* = 8) mice were intravenously injected with 2 IU/mouse of rhFVIII at weekly intervals. One group of HemA mice (purple) received an additional injection of a stimulatory PD-1 antibody (200 μg) intraperitoneally on day 21. (**B**) On day 22, the amount of early apoptotic B cells given as the percentage of annexin V^+^ and Hoechst^–^ FVIII-specific B cells was analyzed after in vitro restimulation with rhFVIII. (**C**) Number of FVIII-specific B cells in spleens of HemA mice treated with a PD-1 stimulatory antibody or not 24 hours after the last injection. *P < 0.05 by unpaired 2-tailed Student’s *t* test.

The fact that PD-L1–expressing Tregs were necessary and sufficient for tolerance toward FVIII raised the question whether PD-1 stimulation alone could trigger apoptosis of FVIII-specific B cells that had been induced to express PD-1 by FVIII injection. To test this hypothesis, we injected a PD-1–specific stimulatory agonist into FVIII-treated HemA mice (Figure 3A). Indeed, more apoptotic FVIII-specific B cells were observed after PD-1 stimulation compared with control mice (Figure 3B) and consistently the total number of viable FVIII-specific B cells was lower (Figure 3C). These findings confirmed that pharmacological PD-1 activation can induce apoptosis of antigen-stimulated FVIII-specific B cells.

### High-dose FVIII treatment induces antigen-specific PD-L1^+^ Tregs and eliminates FVIII^+^ B cells.

**Figure 4:**
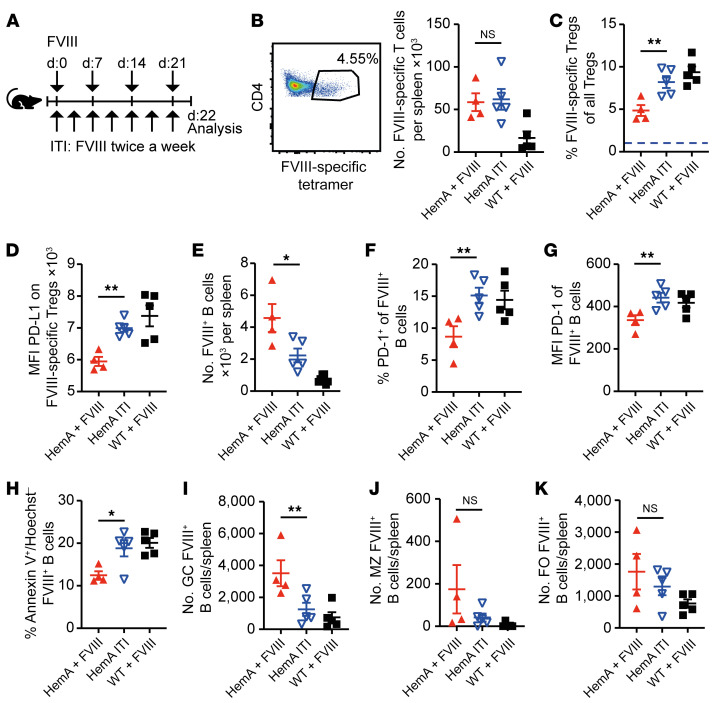
High-dose FVIII treatment expands antigen-specific Tregs and increases their PD-L1 expression. (**A**) Experimental setup for **B**–**K**: 2 IU/mouse of rhFVIII was intravenously injected into HemA mice (red, *n* = 4) or WT mice (black, *n* = 5) at weekly intervals for 3 weeks, and twice a week for the high-dose FVIII regimen (blue, *n* = 5). Experimental setup for high-dose FVIII application regimen, used to induce tolerance (short ITI), and the therapeutic regimen. (**B**) Representative dot plot of CD4^+^ T cells analyzed for FVIII specificity via tetramer staining of rhFVIII-treated mice (left panel) and FVIII-specific CD4^+^ T cell count (right panel). (**C**) Proportion of FVIII-specific Tregs (gated on CD4^+^tetramer^+^CD25^+^CD127^–^ cells) in splenic suspensions of HemA mice after treatment with rhFVIII once or twice a week. (**D**) PD-L1 expression by FVIII-specific Tregs (CD4^+^tetramer^+^CD25^+^CD127^–^ cells) in splenic cells ex vivo. (**E**) Number of FVIII-specific B cells measured by flow cytometry ex vivo after once- or twice-per-week treatment with rhFVIII. (**F**) Proportion of PD-1–expressing FVIII-specific B cells and (**G**) PD-1 MFI on PD-1^+^ FVIII-specific B cells in the spleen on day 22. (**H**) Early apoptotic cells presented as percentage of annexin V^+^ and Hoechst^–^ FVIII-specific B cells after in vitro restimulation with 0.25 μg rhFVIII overnight. (**I**–**K**) Number of FVIII-specific germinal center (GC; **I**), marginal zone (MZ; **J**) and follicular (FO; **K**) B cells in spleens of HemA mice after rhFVIII treatment. Germinal center B cells were identified as B220^+^FVIII^+^GL7^+^ cells, marginal zone B cells as B220^+^FVIII^+^CD93^–^CD21/35^+^IgM^+^IgD^–^ cells, and follicular B cells as B220^+^FVIII^+^CD93^–^CD21/35^–^IgM^–^IgD^+^ cells. *P < 0.05; **P <0.01 by 1-way ANOVA with Bonferroni’s post hoc test. NS, not significant.

The important role of PD-1 in tolerance toward FVIII raised the question of whether this mechanism might also contribute to FVIII tolerance established by ITI protocols that are currently used to eradicate inhibitors in hemophilia A patients. We first wished to clarify whether PD-1 affects the formation of inhibitors. To this end, we established a FVIII protocol mimicking the repetitive high-dose FVIII injections performed during ITI therapy in mice by injecting FVIII twice a week over 21 days (experimental scheme in Figure 4A). Consistent with a previous study showing that inhibitor titers after FVIII injection remain stable over at least 14 weeks in mice (51), we could not yet detect a reduction in FVIII-specific antibodies and inhibitors among the differently treated HemA groups within that small time frame of 3 weeks (Supplemental Figure 9, A and B). However, we noted an increased amount of active FVIII at this early time point (Supplemental Figure 9C), reflecting the recovery of serum FVIII in humans as an early indicator for successful ITI therapy. Furthermore, we established a detection method for FVIII-specific T cells by designing murine MHC II tetramers loaded with a high-binding C2 domain epitope of FVIII, TASSYFTNMFATWSPSKARL (Figure 4B). Since such tetramers detach when cells are permeabilized for intracellular staining, for example for Foxp3, we identified Tregs as CD25^+^CD127^–^CD4^+^ T cells, as previously described (52), and confirmed the expression of *Foxp3* within these cells by quantitative reverse transcription PCR (qRT-PCR) (Supplemental Figure 10). Indeed, we observed in high-dose-FVIII–treated mice a higher number of FVIII-specific Tregs (Figure 4C), and these expressed significantly more PD-L1 (Figure 4D) compared with the HemA group treated once a week (therapeutic regimen). In contrast to the WT control group, the main proportion of FVIII-specific Tregs in high-dose-treated mice were peripherally derived, as evident by the lack of neuropilin-1 expression (Supplemental Figure 11A). Thus, our ITI-like protocol caused an expansion of PD-L1–expressing FVIII-specific induced Tregs, but it did not affect the exhaustion state of FVIII-specific CD4^+^ T cells (Supplemental Figure 11, B–E).

We also examined FVIII-specific B cells in high-dose-FVIII–treated mice, and observed a significant reduction in those B cells (Figure 4E), enhanced PD-1 expression (Figure 4, F and G), and increased apoptosis compared with B cells in mice treated with the standard FVIII regimen (Figure 4H). ITI decreased BCL-2 expression, but did not alter B cell activation as determined by IRF4 expression (Supplemental Figure 12, A–D), supporting apoptosis as the main mechanism of B cell removal. Interestingly, the germinal center B cell subpopulation was most reduced, whereas marginal zone and follicular B cells were somehow less affected by our ITI-like protocol (Figure 4, I–K). These results revealed a tolerized state of FVIII-specific B cells induced by repetitive high-dose FVIII injections.

### Both PD-1 blockade and Treg depletion abolish tolerization of specific B cells after high-dose FVIII.

**Figure 5:**
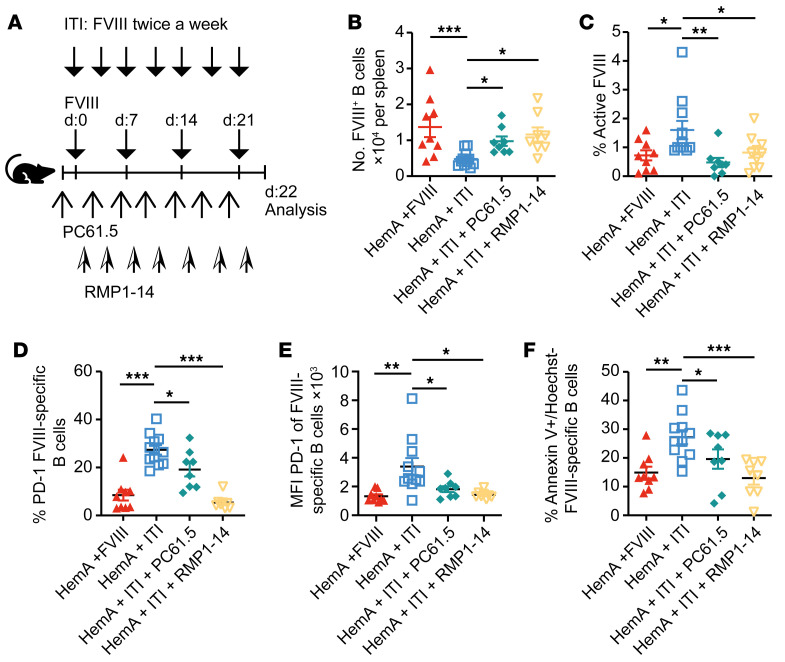
Both PD-1 blockade and Treg depletion abolish immune tolerance induction after high-dose FVIII. (**A**) Experimental scheme for **B**–**F**: 2 IU/mouse of rhFVIII was intravenously injected into HemA (red, *n* = 9) mice at weekly intervals. Immune tolerance induction in HemA mice (blue *n* = 11, green *n* = 8, yellow *n* = 8) was achieved by injecting rhFVIII twice a week. CD25^+^ Tregs were depleted in ITI-receiving HemA mice (green) by the intraperitoneal injection of 250 μg depleting anti-CD25 antibody (PC61.5) 1 day prior to each rhFVIII injection. The PD-1 axis was inhibited in HemA mice (yellow) by injecting an anti–PD-1 inhibitory antibody (RMP1-14) intraperitoneally 3 hours after each rhFVIII treatment. (**B**) Number of FVIII-specific B cells in spleens of HemA mice after treatment with rhFVIII by flow cytometry. (**C**) Percentage of residual active FVIII protein in the plasma of treated mice. (**D**) Percentage of PD-1–expressing FVIII-specific B cells and (**E**) the MFI of PD-1 on FVIII-specific B cells. (**F**) Early apoptotic cells presented as percentage of annexin V^+^ and Hoechst^–^ FVIII-specific B cells after in vitro restimulation with 0.25 μg rhFVIII overnight. *P < 0.05; **P < 0.01; ***P < 0.001 by 1-way ANOVA with Bonferroni’s post hoc test.

Next, we investigated whether our tolerance protocol depended on a functional PD-1 axis. To this end, we depleted Tregs or blocked PD-1 signaling by monoclonal antibodies against CD25 (PC61.5) or PD-1 (RMP1-14), respectively, during the ITI treatment (Figure 5A). Both antibodies abolished tolerance induction, as detected by measuring the increase in FVIII-specific B cells and the reduced fraction of active FVIII in the plasma (Figure 5, B and C). Furthermore, the reduced numbers of FVIII-specific B cells during ITI (Figure 5B) correlated with the increased PD-1 expression (Figure 5, D and E) and with enhanced apoptosis (Figure 5F) of these cells. These findings confirmed PD-1 is required to tolerize FVIII-specific B cells in our ITI protocol.

### High-dose FVIII induces B cell tolerance via PD-1 and Tregs also in mice with existing inhibitors.

**Figure 6:**
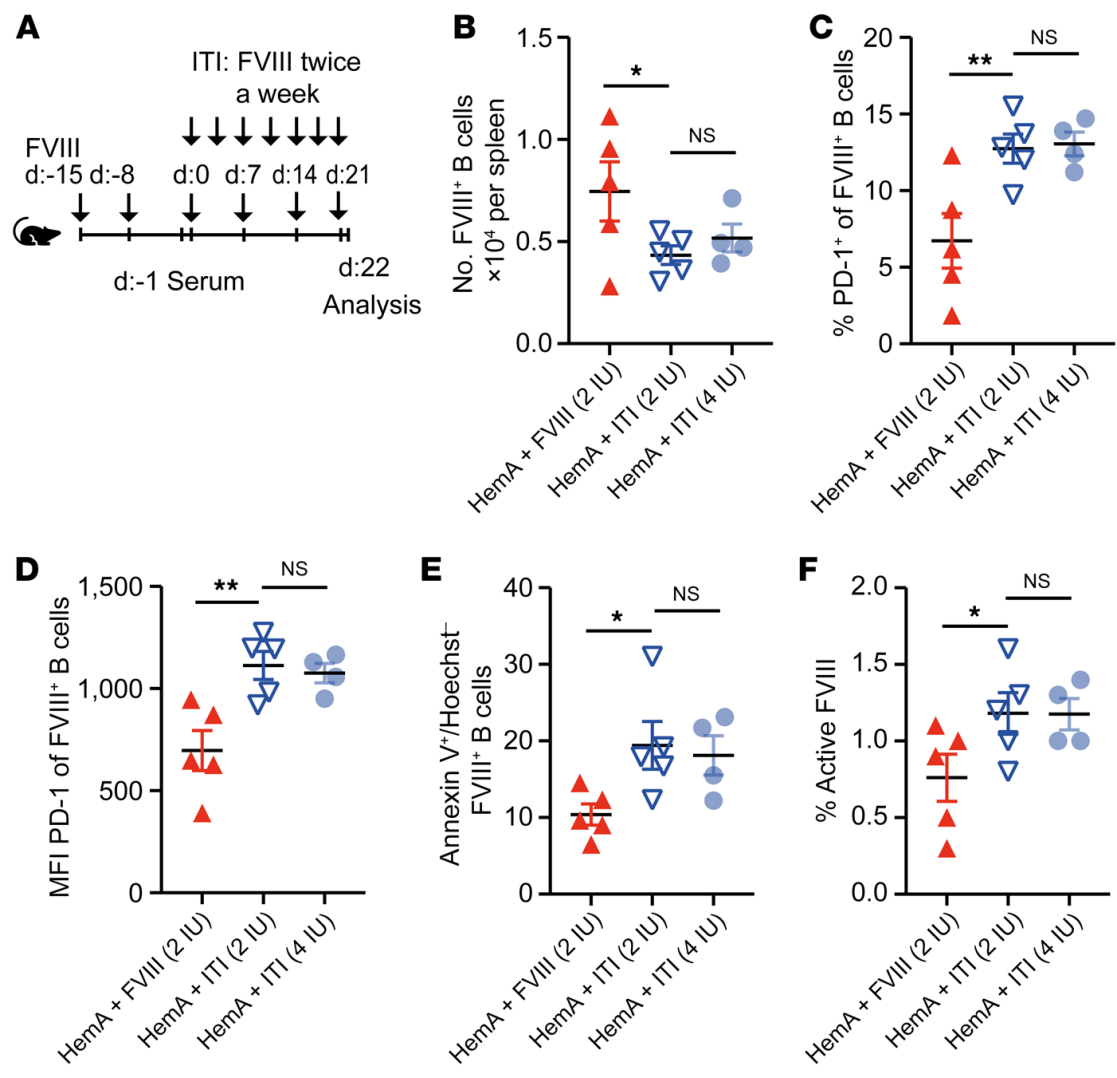
High-dose FVIII induces immune tolerance via PD-1 and Tregs in mice with existing inhibitors. (**A**) Experimental scheme for inhibitor induction prior to ITI. HemA mice were challenged 2 times at a weekly interval with 2 IU rhFVIII per mouse. FVIII-specific IgG titer was determined on day 14 and mice were distributed into the groups to achieve a comparable pretreatment status. Subsequently, HemA mice were immunized again with 2 IU/mouse of rhFVIII therapeutically once a week (red, *n* = 5) or according to the ITI protocol twice a week with 2 IU (blue triangle, *n* = 5) or 4 IU (light blue circle, *n* = 4) per mouse. (**B**) Ex vivo quantification of the number of FVIII-specific B cells in the spleen 22 days after start of ITI by flow cytometry. (**C**) Percentage of PD-1^+^ FVIII-specific B cells and (**D**) the MFI of PD-1 on FVIII-specific B cells analyzed ex vivo. (**E**) Early apoptotic cells are presented as percentage of annexin V^+^ and Hoechst^–^ FVIII-specific B cells after in vitro restimulation with 0.25 μg rhFVIII overnight. (**F**) Percentage of residual active FVIII in the plasma of HemA mice on day 22. *P < 0.05; **P < 0.01 by 1-way ANOVA with Bonferroni’s post hoc test. NS, not significant.

Clinical ITI is performed in hemophilic patients after inhibitors have developed, not before, as in the experiments above. Therefore, we wished to clarify whether our ITI-like protocol was also able to suppress FVIII-specific B cells in mice that already had developed inhibitors. To this end, we treated HemA mice twice with FVIII and measured the levels of inhibitor 14 days after the initial injection. Based on the results, the FVIII-injected mice were equally distributed among all 3 groups to obtain a comparable pretreatment titer (Supplemental Figure 13, A and B). Afterward, one HemA group was challenged again with FVIII in a weekly interval, while the remaining mice received the ITI-like protocol with 2 FVIII injections per week, either with 2 or 4 IU per mouse (Figure 6A). After 22 days, the group that had received the ITI-like protocol with 2 IU FVIII showed significantly reduced numbers of FVIII-specific B cells in the spleen compared with the HemA group that had been treated once a week with 2 IU of FVIII (Figure 6B). Enhancing the dose of FVIII to 4 IU per ITI injection did not further decrease the number of FVIII-specific B cells (Figure 6B). Furthermore, PD-1 expression by FVIII-specific B cells in the ITI-treated group was elevated (Figure 6, C and D). In line with these results, apoptosis induction in FVIII-specific B cells and active FVIII levels were also increased in ITI-treated mice compared with those HemA mice that received the standard FVIII treatment protocol (Figure 6, E and F). These results showed that our ITI protocol operated also after inhibitors had been formed and supported PD-1–mediated apoptosis as the underlying mechanism.

### FVIII-specific B cells of nonhemophilic humans and hemophilia patients under ITI upregulate PD-1.

**Figure 7:**
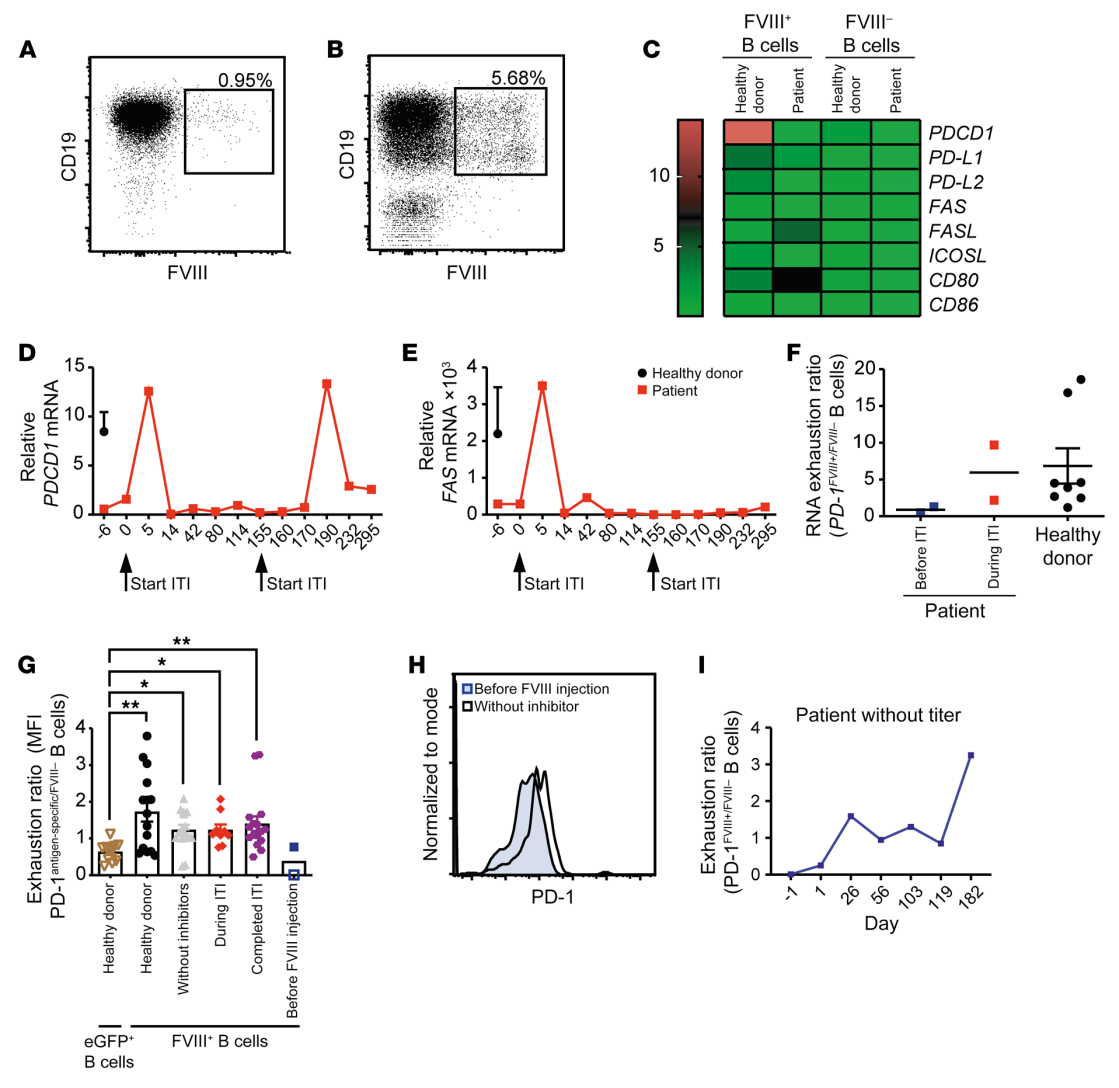
FVIII-specific B cells of healthy humans and hemophilia patients under ITI upregulate PD-1. (**A** and **B**) Gating strategy for sorting of FVIII-specific B cells from human blood samples of a healthy donor (**A**) and a hemophilia A patient before ITI (**B**). CD19^+^ B cells were sorted for their ability to bind to fluorescently labeled rhFVIII protein. mRNA was extracted from sorted FVIII-specific B cells and analyzed by RT-PCR. (**C**) Relative mRNA expression of various inhibitory molecules in FVIII-specific and non–antigen-specific B cells from a hemophilia A patient with inhibitors or from healthy donors. Expression is correlated to 1 healthy individual. (**D**) Relative mRNA expression of *PDCD1* and (**E**) *FAS* in FVIII-specific B cells of 1 hemophilia A patient with inhibitors during ITI. Arrows define the beginning of an ITI cycle. (**F**) RNA exhaustion ratio of *PDCD1* in FVIII-specific B cells and non–antigen-specific B cells in a hemophilia A patient before ITI (blue, *n* = 2), during ITI (red, *n* = 2), and in healthy control donors (black, *n* = 8). (**G**) PD-1 protein expression (exhaustion ratio) of GFP-specific (brown triangles, *n* = 12) or FVIII-specific (all other groups) relative to non–FVIII-specific B cells from healthy donors (black circles, *n* = 15), hemophilia A patients before ITI (blue squares, *n* = 2), during ITI that received a rhFVIII injection less than 24 hours before analysis (gray triangles, *n* = 15), after completing ITI (red diamonds, *n* = 9), or without inhibitor titers (purple hexagon, *n* = 16), determined by flow cytometry. *P < 0.05; **P < 0.01 by 1-way ANOVA with Kruskal-Wallis post hoc test. (**H**) PD-1 expression on FVIII-specific B cells from the patient displayed in **G** as an open blue square (x axis) before (blue-filled area) and after rhFVIII injection on day 182 (unfilled area) presented as histogram. (**I**) Development of the exhaustion ratio of FVIII-specific B cells over 6 months.

Next, we asked whether the B cell–inhibitory mechanism identified in mice was also operative in humans. To this end, we first adapted our fluorescent-FVIII-based flow cytometric staining protocol for FVIII-specific CD19^+^ B cells to the human system, aiming to sort such B cells from healthy volunteers or hemophilia A patients for analytical comparison. However, because of the paucity of such B cells (Figure 7, A and B), 50 mL of blood was required to obtain sufficient mRNA. Therefore, we could not use this method to analyze hemophilia A patients before ITI, because these patients are usually very young at diagnosis and cannot donate that large an amount of blood. However, one adult hemophilia A patient in our outpatient clinic had started developing inhibitors comparatively late in life and underwent at the age of 40 ITI following a modified Malmö protocol (53, 54). That patient volunteered to provide that amount of blood, before and at various time points during ITI.

FVIII-specific B cells of healthy (FVIII-tolerant) volunteers expressed higher *PDCD1* (which encodes PD-1) mRNA levels than did their non–FVIII-specific B cells (Figure 7C), the majority of which can be assumed to be specific for foreign antigens. In contrast to *PDCD1*, FVIII-specific B cells of healthy volunteers expressed either lower or similar amounts of *FAS* (Figure 7C), or of the coinhibitory molecule *PD-L1*, *PD-L2*, or *FASL* (Figure 7C) compared with non–FVIII-specific B cells, suggesting that *PDCD1* is involved in tolerance against FVIII also in healthy humans.

Before ITI, the population of FVIII-specific B cells in our patient was much larger than in healthy donors (Figure 7, A and B), reflecting the absence of tolerance against FVIII. Before ITI, neither *PDCD1* nor *FAS* mRNA expression was detectable in sorted FVIII-specific B cells (Figure 7, D and E), consistent with the inactivity of both signaling pathways in B cells specific for a foreign antigen. Already a few days after the start of ITI treatment, an increase in *PDCD1* and *FAS* expression (Figure 7, D and E), but not of the other coinhibitory molecules under examination (Supplemental Figure 14, A–C), was detectable on FVIII-specific B cells of our patient. This is likely explained by the canonical induction of FAS and PD-1 after B cell receptor signaling (33, 55).

In our patient, ITI had to be interrupted after 1 week because of a respiratory infection. When that infection was overcome, another cycle of ITI was initiated 160 days later. Again, a transient *PDCD1* increase in FVIII-specific B cells was detectable, which, importantly, remained elevated after the actual treatment cycle at least until day 286 (Figure 7D). However, a second increase was not detected for *FAS* expression (Figure 7E). To compare PD-1 expression between our patient and healthy individuals, we calculated the ratio of *PDCD1* RNA expression by FVIII-specific versus non–FVIII-specific B cells, here referred to as an “exhaustion ratio” in analogy to this function of PD-1 in T cells (36, 37). During ITI, this ratio increased to levels comparable to healthy donors (Figure 7F), suggesting that ITI engaged the PD-1 pathway in human FVIII-specific B cells.

These findings encouraged us to include more patients in our study. Focusing on PD-1 as a tolerance mediator candidate allowed us to move from mRNA analysis via RT-PCR to protein analysis via flow cytometry, which does not require cell sorting and therefore can be performed with much less blood, for example 1.5 to 5 mL. This gave us the opportunity to study children with hemophilia A. We determined the exhaustion ratio from flow cytometric PD-1 expression data of B cells, analogous to the mRNA exhaustion ratio described above. To compare with B cells specific for a foreign antigen, we chose GFP, and FVIII in healthy individuals as an example for autoreactive B cells. Consistent with our murine data, the exhaustion ratio in healthy donors was higher in FVIII-specific B cells compared with GFP-expressing B cells (Figure 7G). Comparably elevated was the exhaustion ratio in hemophilia A patients that had not developed inhibitors (Figure 7G). Likewise, patients that previously had developed inhibitors and underwent successful ITI also showed an elevated exhaustion ratio, both during therapy and after its completion (Figure 7G), suggesting that successful ITI establishes PD-1–mediated immune tolerance in hemophilia A patients.

Additionally, we had the rare opportunity to examine 2 babies with hemophilia A before they received the first therapeutic FVIII injection. Their exhaustion rate was very low in one case and comparable to GFP-positive B cells in the other case (Figure 7G), consistent with the phenotype of B cells specific for a foreign antigen. We followed the course of one of the patients with a very low exhaustion rate over half a year of FVIII treatment. During that time, that patient did not develop inhibitors, so ITI did not need to be performed. Nevertheless, PD-1 expression on day 182 was higher on FVIII-specific B cells compared with day 1 (Figure 7H). Consistently, the exhaustion rate of his FVIII-specific B cells continually increased over time, indicating increasing susceptibility to control by PD-L1^+^ Tregs (Figure 7I).

### PD-1 stimulation induces apoptosis in human FVIII- and FIX-specific B cells.

**Figure 8:**
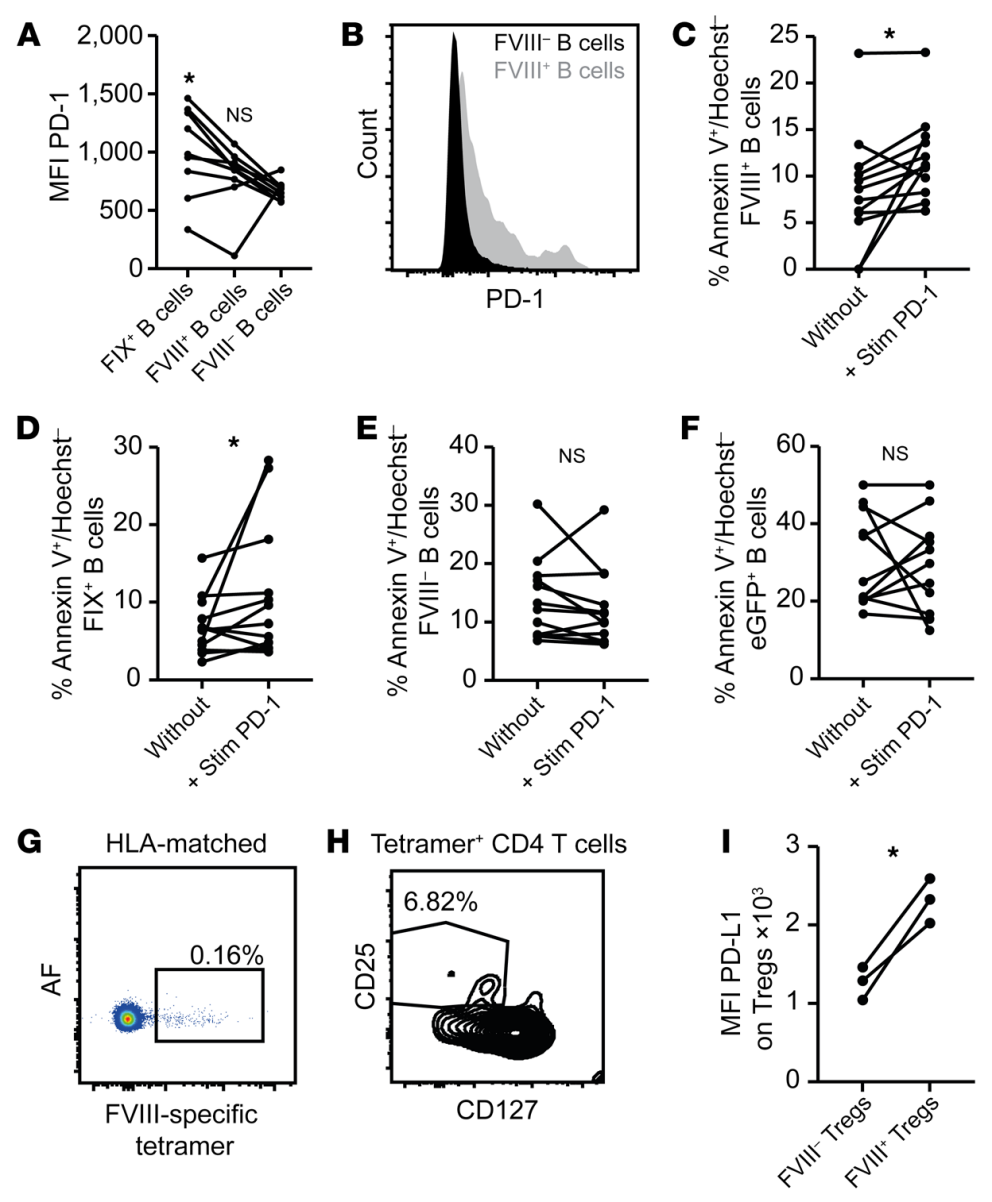
PD-1 stimulation induces apoptosis in human FVIII- and FIX-specific B cells. (**A**) MFI of PD-1 expression by FVIII- and FIX-specific and nonspecific B cells from 9 healthy donors detected by flow cytometry. *P < 0.05 by 1-way ANOVA with Dunn’s post hoc test. NS, not significant. (**B**) Example of PD-1 expression by FVIII-specific B cells from 1 representative donor. (**C**–**F**) Percentage of apoptotic FVIII-specific (**C**), FIX-specific (**D**), non–FVIII-specific (**E**), and GFP-specific (**F**) B cells without and after incubation with a PD-L1 chimeric protein that specifically stimulates PD-1 in vitro. (**G**) The presence of FVIII-specific CD4^+^ T cells was analyzed in blood samples of an HLA-matched patient sample (*n* = 3) who had undergone successful ITI. (**H**) The percentage of CD25^+^CD127^–^ (Tregs) of HLA-matched tetramer^+^CD4^+^ cells is depicted as a representative example. (**I**) The geometric MFI of PD-L1 was analyzed on FVIII-specific CD4^+^ HLA-matched Tregs versus nonspecific Tregs from the same patient blood sample. *P < 0.05 by 1-way ANOVA with Dunn’s post hoc test (**A**) or paired, 2-tailed Student’s *t* test (**C**–**F** and **I**). NS, not significant.

Next, we aimed to demonstrate that PD-1 can induce immune tolerance of human B cells specific for coagulation factors. FVIII-specific human B cells of healthy donors expressed slightly more PD-1 than nonspecific B cells (Figure 8, A and B). This was also seen in B cells specific for coagulation factor IX (FIX) (Figure 8A), which is mutated in hemophilia B, another X-linked coagulopathy that is much rarer than hemophilia A but causes similarly serious symptoms and is also associated with a risk of developing inhibitors (4). When we cultured these B cells with a PD-1–stimulating agonist, in order to mimic effects of PD-L1^+^ Tregs, both FVIII- and FIX-specific B cells responded by undergoing apoptosis (Figure 8, C and D). By contrast, FVIII-negative B cells did not do so (Figure 8E), consistent with their lower PD-1 expression (Figure 8, A and B). Their response resembled that of B cells specific for the foreign antigen GFP, which also did not undergo apoptosis upon PD-1 stimulation (Figure 8F).

Finally, we wished to demonstrate that FVIII-specific Tregs exist in humans. To this end, we designed MHC II tetramers that can bind the FVIII peptide, TLFFQNGKVKVFQGNQDSFT, to stain FVIII-specific CD4^+^ T cells in patients expressing the HLA-DR15.01 allele. When we used these tetramers in an HLA-DR15.01^+^ hemophilia A patient after successful ITI, we indeed detected a distinct subset of FVIII-specific CD4^+^ T cells, as opposed to an HLA-mismatched control patient (Figure 8G and Supplemental Figure 15). Using standard staining protocols for human Tregs based on CD25 and the lack of CD127 (52), we indeed detected a proportion of Tregs among these FVIII-specific CD4^+^ T cells (Figure 8H; 6.79% ± 1.1%). These Tregs expressed higher levels of PD-L1 compared with tetramer-negative Tregs originating from the same patient (Figure 8I). These findings demonstrated the existence of PD-L1–expressing Tregs in humans and supported their role in PD-1–mediated tolerance to FVIII.

## Discussion

Neutralizing antibodies are the most prevalent and serious complication of hemophilia therapy (4–6, 9). The immune mechanisms governing inhibitor formation are not well understood. Protocols to induce tolerance toward infused coagulation factors are expensive, burdensome, and not always effective. Furthermore, the mechanisms underlying such tolerance induction are unclear. Here we propose a pathophysiological link between inhibitor-producing B cells and Tregs, which is mediated via PD-L1/PD-1 signaling. Our findings suggest that this molecular mechanism is critical also for induction of tolerance to FVIII under nonhemophilic conditions, as well as for establishing and maintaining tolerance toward FVIII during ITI in hemophilia A patients. We furthermore suggest that ITI may be related to a long known immunological phenomenon termed “high zone tolerance” (16). Thus, our findings may not only be relevant for understanding immune tolerance to FVIII, but to soluble high-dose antigens in general.

Our second finding is that also ITI protocols, such as the Bonn and Malmö protocols, may operate through PD-L1^+^ Treg–induced B cell apoptosis. We demonstrated this by establishing a murine model that mimics important aspects of the ITI protocols for treating humans. We noted that both, PD-1 blockade and Treg depletion, significantly impaired tolerance induction in our model. Tregs have been shown to suppress auto-Ab formation (56, 57), and are being discussed as a potential therapeutic tool to prevent FVIII inhibitor formation (58, 59). Since Tregs are widely recognized to suppress other T cells, it is usually assumed that Tregs suppress B cells indirectly by inhibiting fTh cells (60). However, there is evidence by us and others that Tregs can suppress B cells also directly (32). We have previously used mice expressing transgenic tissue-restricted model antigens to demonstrate that PD-1 on autoreactive B cells is critical for such suppression (31). In our present study, we show that also inhibitor-producing B cells in HemA mice expressed PD-1, initially at low levels, but upregulated it after recognizing their cognate antigen. Subsequently, they underwent apoptosis upon direct encounter with PD-L1^+^ Tregs that had been induced by ITI, identifying PD-1 as an important checkpoint also in allospecific antibody production. We did not observe exhaustion signs in FVIII-specific CD4^+^ T cells, nor altered expression of PD-L1 and PD-L2 (data not shown). This does not rule out any role for Th cells, but supports the conclusion that Treg-mediated B cell apoptosis is a main mechanism by which ITI operates.

In our experiments, PD-1 blockade seemed to be somewhat more effective than Treg depletion. This may be explained either by incomplete Treg depletion, or it may indicate that the PD-1–blocking antibody may target other relevant immune cells, in particular fTh cells, which are well known to express PD-1. Their inhibition might thus suppress inhibitor formation indirectly. However, our findings suggest that direct B cell suppression through PD-1 is sufficient to reduce inhibitor formation to a clinically relevant extent.

It was recently reported that B cell–activating factor (BAFF) stimulated the production of FVIII inhibitors and/or FVIII-specific B cells (61). Given that BAFF stimulation can activate B cells and upregulate their CD80 and PD-L1 expression (62), it appears conceivable that BAFF might negatively regulate their PD-1 expression and thereby promote their survival. Future studies may address this notion.

It has been previously shown that the increased availability of FVIII during ITI initially drives inhibitor production, followed by a subsequent decline after about 4 to 8 weeks in humans (14). Such a delay has been noted also in mice, but it took much longer (>14 weeks) (51). The delay as such validates the mouse model, but its length precludes the use of inhibitor titers as an experimental readout within common experimental time frames. This problem has hindered scientific progress in experimental inhibitor research. We could overcome this obstacle by monitoring inhibitor-producing B cells via flow cytometry. Their deletion served as a surrogate readout, since it is reasonable to assume that inhibitors will decline if the inhibitor-producing cells are lost. Despite this caveat, our findings suggest that ITI operates at least in part by inducing FVIII-specific Tregs that promote apoptosis of FVIII-specific B cells, thereby stopping inhibitor formation. The inhibitors produced prior to B cell apoptosis are likely partially bound to injected FVIII, and the rest will be cleared with time, resulting in the above-mentioned delayed inhibitor disappearance. Another contributing factor is the time required for inducing Tregs, which in our experience requires 3 to 4 weeks in mice (data not shown), consistent with studies by others (46). This interpretation is further supported by the increase in active FVIII in our murine ITI model after 3 weeks of FVIII treatment, which heralds the decrease in inhibitors. The delayed decrease in inhibitors in humans under ITI may further be explained by long-lived plasma cells residing in bone marrow survival niches at the time of ITI initiation. Further studies are needed to clarify whether these cells can be eliminated by combining ITI and/or applying PD-1–stimulating antibodies over a longer time period. If not, PD-L1^+^ Tregs may be less effective in patients with very high inhibitor titers, in which plasma cells likely contribute to inhibitor production.

Studying the immune mechanisms in humans with hemophilia A with inhibitors is difficult because treatment usually starts during childhood, which entails ethical problems, as appreciable amounts of blood are needed for flow cytometric analysis. We had the unique opportunity to study an adult patient who developed inhibitors comparatively late in life and underwent his first ITI at the age of 40 years. FVIII-specific B cells were detectable in that patient’s blood and among the checkpoint molecules known, only PD-1 expression after ITI therapy followed the expression pattern we had observed in our mouse models. PD-1 is generally upregulated after T cell activation and remains expressed in situations of T cell exhaustion, for example during chronic viral infection or in tumors (36–38). Hence, PD-1 has been described as an activation marker on human B cells (63). Based on this, we hypothesized that persistent PD-1 expression after ITI might indicate that FVIII-specific B cells are exhausted and killable, and thereby heralds ITI success. Focusing our analysis of PD-1 reduced the amount of blood needed for flow cytometry, so that small children undergoing ITI became available for analysis. We found that the PD-1 exhaustion ratios of FVIII-specific B cells in patients that did not develop inhibitors, or in patients during or after successful ITI, were comparable to healthy individuals, but not to ratios in B cells specific for a foreign antigen. We also had the opportunity to follow the clinical course of 1 very young hemophilia A patient before and during FVIII treatment. That patient did not develop inhibitors and the PD-1 exhaustion ratio of his FVIII-specific B cells continually increased, even above values in healthy individuals, consistent with regained immunological control. Such FVIII-specific B cells will undergo apoptosis when PD-1 is ligated, as we formally demonstrated by coculture experiments. These findings indicated that PD-1 on human B cells is functional and capable of inducing tolerance, for example when triggered by PD-L1^+^ Tregs that had been induced by ITI.

The link between PD-1 and ITI of FVIII-specific B cells is consistent with the idea that ITI renders FVIII-specific B cells susceptible to the control of the immune system via antigen-specific PD-L1^+^ Tregs (32). These B cells likely present FVIII-derived peptides on their surface, so that they can be recognized by and eliminated by PD-L1^+^ Tregs that had been previously induced by ITI. Indeed, using specifically designed MHC II–peptide tetramers, we were able to document the induction of antigen-specific PD-L1^+^ Tregs in our murine ITI model and their existence in hemophilia patients after successful ITI. To the best of our knowledge, our study is the first to identify such Tregs, which offers new opportunities for basic research, diagnosis, and therapy of hemophilia. Antigen-specific Tregs are frequently discussed as tools to regulate unwanted immune responses, such as those in hemophilia A patients with inhibitors (23). However, until now tools to directly study FVIII-specific Tregs have been lacking. The MHC II tetramers used here may advance research by allowing such direct analysis of FVIII-specific Tregs. Further studies may characterize such Tregs and define their potential to improve the treatment of hemophilia A patients with inhibitors.

MHC II tetramers are expensive, difficult to use, and only applicable in patients expressing a specific MHC allotype. The PD-1 exhaustion ratio we described is MHC independent and also less expensive. Such a diagnostic tool is desirable as a prognostic biomarker of ITI success, to assist in the decision whether an expensive ITI should be continued or not, or, in case of success, to plan tapering the FVIII dose. It may also serve as a risk assessment tool to monitor inhibitor development, to decide whether switching from clotting factor substitution therapy to alternative treatment, such as FIX-FX–bispecific antibodies (12), should be considered. Larger studies are warranted to assess the clinical value of our assay.

Our findings may aid future therapeutic attempts to treat inhibitor-complicated hemophilia by transferring PD-L1^+^ antigen-specific Tregs through CAR technology (64–67). Finally, the mechanism identified here may operate also in other genetic diseases where the supplementation of recombinant proteins is complicated by inhibitor formation, such as Fabry or Pompe disease (68–70).

## Methods

### Human patients.

Human blood samples were obtained from 34 patients with hemophilia A and 17 healthy individuals. Thirty-two hemophilia patients suffered from a severe phenotype characterized by residual FVIII activities (FVIII clotting activity [FVIII:C] below 1%). Two patients were moderately affected (FVIII:C between 1% and 5%). Fourteen patients had high titer (5 Bethesda units [BU] or higher) neutralizing antibodies against FVIII and another 5 patients had a history of low-titer (below 5 BU) neutralizing inhibitors measured by the Nijmegen Bethesda assay. Fifteen patients lacked neutralizing antibodies against FVIII until blood was drawn for analysis. Fourteen patients had been exposed to FVIII, but 2 did not receive FVIII before blood was drawn (drug-naive control). Of the 19 patients with a history of inhibitors, 12 had completed ITI treatment successfully and 5 patients had completed ITI with partial success. One patient had failed in a first ITI attempt and had started a second ITI treatment cycle when blood was drawn. For 1 adult patient with severe hemophilia A and a long history of high-titer neutralizing antibodies against FVIII, ITI was monitored longitudinally. That patient suffered from an intron 22 inversion and lacked FVIII secretion. ITI was performed according to a modified Malmö protocol (high-dose FVIII, immune suppression with mycophenolate mofetil and steroids, i.v. IgG) (53, 54). For this patient, samples were obtained before start of the first ITI treatment cycle on day –6 and during this first ITI cycle (days 0, 5, 14, and 42). After the first ITI cycle had to be stopped on day 49, further samples were drawn on day 80 and day 114. A second ITI cycle was started on day 154 and blood was obtained on days 155, 159, 171, and 190 (corresponds to days 1, 5, 17, and 36 of second ITI cycle). This second ITI cycle was stopped on day 194. Follow-up post-ITI samples were taken on day 232 and day 295 (days 78 and 141 of second ITI cycle). We also followed a baby patient whose mother carried a missense mutation in exon 1, c.2T>G (p.Met1Arg) over half a year.

### Human blood samples.

PBMCs were obtained from human blood by centrifugation in a gradient of Percoll (PromoCell). Cells were collected and stained as described in *Flow cytometry and cell sorting*. To stain for apoptosis, human lymphocytes were cultured with 5 μg/mL recombinant human PD-L1–Fc chimera protein (R&D Systems, 156-B7) overnight.

### Mice.

All mice (C57BL/6, C57BL/6 × 129S, HemA [B6;129S-*F8^tm1Kaz^*, ref. 42], *Pd-l1^–/–^*, Foxp3-LuciDTR) were bred and maintained under specific pathogen–free conditions at the central animal facility of the University clinic of Bonn. The HemA, C57BL/6, and C57BL/6 × 129S mutant mouse strains were from The Jackson laboratory and used at 8–14 weeks of age at the beginning of the experiments. 129S-F8^tm1Kaz^ were on a C57BL/6 × 129S F1 genetic background and were compared to C57BL/6 × 129S F1 control mice. *Pd-l1^–/–^* and Foxp3-LuciDTR mice were maintained internally on a C57BL/6 background and compared to C57BL/6 mice.

### Treatment with FVIII.

Mice were treated with weekly i.v. injections of 2 IU recombinant human FVIII protein (rhFVIII, Kogenate, Bayer) diluted in PBS for a total of 4 weeks (days 0, 7, 14, and 21). For ITI treatment, mice received the same doses of rhFVIII twice a week. The injection sites were closed by electric cauterization to prevent bleeding.

### Reagents.

CD25^+^ cells were depleted by injecting 250 μg of anti-CD25 antibody (PC61.5, BioXCell) i.p. 1 day prior to each rhFVIII injection. Foxp3^+^ cells in Foxp3-LuciDTR mice were depleted by injecting 15 ng/g mouse diphtheria toxin (DTX, Merck) i.p. on days –1 and 0 of the respective experiment. Inhibitory antibodies (250 μg) RMP1-14 (BE0146, BioXCell) or 9H10 (BE0131, BioXCell) were injected i.p. twice a week to block PD-1 or CTLA4 signaling, respectively. Stimulatory PD-1 agonist (200 μg; ProSci, catalog 90-431) was injected i.p. once.

### Blood collection.

For determining active FVIII levels and anti-FVIII antibody titers, blood samples were obtained by cardiac puncture. To receive serum, the samples were kept at room temperature for 30 minutes. The clot was removed and samples centrifuged for 7 minutes at 18,506 *g* and room temperature. Serum was transferred into a fresh tube. For evaluating active FVIII, freshly harvested blood was diluted 9:1 with 0.1 mol/L sodium citrate (Merck), kept on ice, and centrifuged for 20 minutes at 300*g* and room temperature. Plasma was transferred into a fresh tube and subsequently stored at –20°C.

### Active FVIII measurement.

Active FVIII in citrate-buffered plasma of mice was determined using COATEST SP4 FVIII-82 4094 63 (Chromogenix), following the manufacturer’s instructions. Coagulation reference was obtained from Technoclone.

### ELISA.

Total anti-FVIII antibody titers in the blood serum of rhFVIII-treated mice were measured by ELISA. High-binding microplates (Greiner Bio-One) were coated with 1.25 μg/mL rhFVIII diluted in coating buffer (50 mM NaHCO_3_ in PBS, Carl Roth) at 4°C overnight. Nonspecific binding sites were blocked for 1 hour at room temperature using 1% bovine serum albumin (BSA, Merck) in PBS. Starting with a 1:20 dilution, plates were subsequently incubated with a serial dilution of serum samples at 4°C overnight. To detect FVIII-specific IgG antibodies bound to the immobilized rhFVIII protein, the plates were incubated with Biotin-SP–conjugated AffiniPure Goat Anti–Mouse IgG (Jackson Immuno Research) at a 1:10,000 dilution for 2 hours at room temperature. Subsequently, the plates were incubated with streptavidin-labeled horseradish peroxidase (Natutec) at a dilution of 1:5,000 for 1 hour at room temperature. σ-Phenylene diamine dihydrochloride (Thermo Fisher Scientific) and H_2_O_2_ (Carl Roth) were used as substrates to detect immobilized anti-FVIII antibodies. The reaction was stopped using 1 M H_2_SO_4_ (Carl Roth).

Antibody titers were expressed as the highest dilution of plasma samples showing a positive result (optical density > 0.2) in the ELISA assay. The starting dilution was 1:20; if this was not sufficient to detect a signal, the dilution was further decreased.

### Bethesda assay.

Inhibitors were measured using a Bethesda assay (Technocone, FVIII Inhibitor Reagent Kit) according to the manufacturer’s instructions (71). Venous blood was mixed with Sodium Citrate Solution (1:10). All citrated plasma test samples were incubated at 56°C for 10 minutes to inactivate the low amount of residual FVIII. After inactivation, the plasma samples were diluted in Imidazole Buffer. The dilution was established according to the following test group classification: (a) without FVIII inhibitors, (b) weak FVIII inhibitors, or (c) strong FVIII inhibitors. The diluted samples were incubated with FVIII Normal Plasma (containing 1 IU/mL FVIII) at 37°C for 2 hours, according to the the Factor VIII Inhibitor Reagent Kit (Technoclone). Normal Plasma was incubated with FVIII Inhibitor Plasma or FVIII Inhibitor Free plasma, as a negative or positive reference, respectively. The residual activity of FVIII contained in the Normal Plasma after incubation with citrated plasma or inhibitor references was detected by chromogenic assay and calculated by dividing the FVIII value of test sample by the FVIII value in Normal Plasma (nonincubated control). The BU value was obtained with the following equation, according to the instruction manual: BU = (2 − log[residual FVIII activity])/0.30103.

The final value of BU/mL was calculated by multiplying BU values by the dilution factor.

### ELISpot.

For enumerating anti-FVIII antibody–secreting cells, single-cell suspensions of splenocytes were prepared as described above and resuspended in X-Vivo medium (Lonza) supplemented with 10% fetal calf serum (FCS; Thermo Fischer Scientific), 100 U/mL penicillin, 100 U/mL streptomycin, and 5.5 × 10^–5^ M β-mercaptoethanol (Merck Millipore). ELISPOT MultiScreen plates (Merck Millipore) were coated with 1.25 μg/mL rhFVIII diluted in coating buffer (50 mM NaHCO_3_ in PBS, Carl Roth) at 4°C overnight. Cells were plated in a serial dilution starting with 2 × 10^6^ cells and incubated for 4 hours at 37°C and 5% CO_2_. To detect antigen-specific B cells secreting anti-FVIII antibodies that bound to the membrane, the plates were incubated with Biotin-SP–conjugated AffiniPure Goat Anti–Mouse IgG (Jackson Immuno Research) at a 1:10,000 dilution for 2 hours at room temperature. Subsequently, the plates were incubated with streptavidin-labeled horseradish peroxidase (Natutec) at a dilution of 1:5,000 for 1 hour at room temperature. For the visualization of the antibody spots, 3-amino-9-ethylcarbazole (Merck) diluted in 0.1 M sodium acetate was used as substrate.

### Preparation of spleen cells.

Spleens were harvested 1 day after the last rhFVIII injection and passed through a 100 μm nylon cell strainer (Greiner Bio-One) to obtain single-cell suspensions in PBS. Subsequently, cells were centrifuged for 5 minutes at 453 *g* at 4°C and cleared from erythrocytes using a hypotonic red cell lysis buffer (146 mM NH_4_Cl, 10 mM NaHCO_3_, and 2 mM EDTA). Hemolysis was stopped with RPMI 1640 (Life Technologies) media supplemented with 2% FCS. After centrifugation, the cells were finally resuspended in the medium required for the respective analysis.

### Restimulation of splenocytes with rhFVIII.

Splenocytes were isolated as described above and resuspended in X-Vivo medium (Lonza) supplemented with 10% FCS, 100 U/mL penicillin, 100 U/mL streptomycin, and 5.5 × 10^–5^ M β-mercaptoethanol. Approximately 1 × 10^7^ splenocytes/well were plated on a 96-well plate (TPP, Sigma-Aldrich) and restimulated with 2.5 μg rhFVIII at 37°C and 5% CO_2_ overnight. For B cell–Treg coculture of naive splenocytes, 0.5 × 10^6^ splenocytes and Tregs were coincubated at 37°C and 5% CO_2_ overnight in a 48-well plate (TPP) in the presence of 2.5 μg rhFVIII (Kogenate, Bayer) where indicated.

### Isolation and adoptive transfer of primary Tregs.

Single-cell suspensions from spleens were generated as described above. CD4^+^CD25^+^ Tregs were further purified using the murine CD4 CD25 Regulatory T Cell Isolation Kit from Miltenyi Biotec following manufacturer’s instructions. The purity of the transferred CD4^+^CD25^+^ Tregs was controlled with the help of additional intracellular Foxp3 staining, and was 84% to 90%. Cells (1 × 10^6^) in PBS were adoptively transferred into acceptor mice by i.v. injections.

### Isolation of B cells for in vitro coculture.

To isolate antibody-secreting B cells, splenic single-cell suspensions from rhFVIII-injected HemA mice were generated as described above. Subsequently, cells were centrifuged for 5 minutes at 1,500 rpm at 4°C and cleared from red blood cells by hemolysis using a hypotonic red cell lysis buffer (146 mM NH_4_Cl, 10 mM NaHCO_3_, and 2 mM EDTA). Hemolysis was stopped with RPMI 1640 (Life Technologies) media supplemented with 2% FCS. After centrifugation, cells were handled according to the manufacturer’s instructions (Miltenyi Biotec, Pan B cell isolation Kit II). Finally, 0.3 × 10^5^ preactivated B cells were cocultured with 0.5 × 10^5^ WT Tregs (isolated as described above). To block PD-1 signaling, an inhibitory PD-1 antibody (RPM1-14; 40 μg/mL) was added to the coculture. Coculture was performed in X-Vivo medium (Lonza) supplemented with 10% FCS, 100 U/mL penicillin, 100 U/mL streptomycin, and 5.5 × 10^–5^ M β-mercaptoethanol in a 96-well plate (TPP) and restimulated with 2.5 μg rhFVIII at 37°C and 5% CO_2_ overnight.

### Flow cytometry and cell sorting.

Murine splenocytes and human blood cells were centrifuged and stained in PBS supplemented with 0.1% BSA and 0.1% sodium azide (Carl Roth) for 30 minutes at 4°C using fluorochrome-labeled monoclonal antibodies. Nonspecific binding sites were blocked with Fc Block (Grifols). Dead cells were excluded using 7-AAD (Thermo Fisher Scientific). To identify FVIII- or FIX-specific B cells, soluble rhFVIII (Kogenate, Bayer) or rhFIX (Octanine, Octapharma) was conjugated to Alexa Fluor 647 fluorochrome with a commercial kit (Invitrogen) according to the manufacturer’s instructions. Unbound fluorophore was removed by spin column purification (Biotium). Subsequently, protein concentration was determined by Nanodrop (Thermo Fisher Scientific) and 5 × 10^6^ cells were stained with 1.25 μg/mL labeled protein. Apoptosis was determined using the annexin V–FITC Apoptosis Detection Kit (eBioscience), together with Hoechst 33342 (Molecular Probes), following the manufacturers’ instructions. GFP-specific B cells were identified using 20 μg EGFP protein (ab84191, Abcam) for surface staining. For intracellular staining, cells were fixed and stained using the Foxp3/Transcription Factor Staining Buffer Set (eBioscience) and following the manufacturer’s instructions. To determine absolute cell numbers, 1 × 10^5^ CaliBRITE APC beads were added before flow cytometry as internal control. Samples were analyzed and sorted using a FACSCanto II, LSRFortessa, or FACSAria III (BD Biosciences). Results were analyzed with FlowJo v.10.5.3 software (FlowJo, LLC).

Antibodies (and their clone numbers) against the following proteins from BioLegend (unless specified otherwise) were used for murine cells: B220 (RA3-6B2), CD86 (GL-1), CD4 (GK1.5), Foxp3 (150D), CD25 (PC61), neuropilin-1 (3E12), Helios (22F6), GL7 (GL7), CD93 (AA4.1), IgD (11-26c2A), IRF4 (IRF4.3E4), Bcl-2 (BCL/10C4), neuropilin-1 (3E12), FVIII-Tetramer (catalog 4118, ProImmune), PD-1 (J43, eBioscience), Fas (15A7, eBioscience), CD21/35 (7G6, BD Biosciences), IgM (R6-60.2, BD Biosciences), and CD80 (3H5, BD Biosciences).

Antibodies (and their clone numbers) against the following proteins from BioLegend (unless specified otherwise) were used for human cells: PD-1 (EH12.2H7), CD80 (2D10), CD3 (UCHT1), CD11c (3.9), CD66b (G10F5), CD4 (OKT4), CD8 (RPA-T8), CD20 (2H7), CD127 (A019D5), CD19 (HIB19, eBioscience), CD14 (61D3, eBioscience), and FVIII-Tetramer (catalog 3140, ProImmune).

### Tetramer design.

MHC class II tetramers were designed to identify FVIII-specific CD4^+^ T cells in mice and humans. Peptide sequences located within the A2 and C2 domain of human FVIII were analyzed according to their predicted MCH class II binding affinity using the IEDB Analysis Resource (http://tools.iedb.org/main/). Based on the sequences with the highest binding affinity, murine (IAb/TASSYFTNMFATWSPSKARL; FVIII_2210–2229_) and human (TLFFQNGKVKVFQGNQDSFT; FVIII_2291–2310_) MHC class II (HLA-DR15.1) tetramers were generated in collaboration with ProImmune. Both tetramers were labeled with phycoerythrin (PE) and used for flow cytometry.

### RNA extraction from human cells and quantitative PCR.

Total RNA was isolated using an RNeasy kit (Qiagen) and reverse transcription was performed using the High-Capacity RNA-to-cDNA kit (Thermo Fisher Scientific). Real-time quantitative PCR was performed on LightCycler 480II (Roche). The following primers were purchased from Invitrogen and used for quantitative PCR: *PD-L1* (fwd 5′-TGTACCACGTCTCCCACATAACAG-3 rev 5′-ACCCCACGATGAGGAACAAA-3′), *PD-L2* (fwd 5′-TGACCCTCTGAGTTGGATGGA-3′ rev 5′-GCCGGGATGAAAGCATGA-3′), *PDCD1* (fwd 5′-AAGCTTATGTGGGTCCGGC-3′rev 5′-GGATCCTCAAAGAGGCC-3′), *FASL* (fwd 5′-CGGTGGTATTTTTCATGGTTCTGG-3′rev 5′-CTTGTGGTTTAGGGGCTGGTTGTT-3′), and *FAS* (fwd 5′-TCTGGTGCTTGCTGGCTCAC-3′rev 5′-CCATAGGCGATTTCTGGGAC-3′).

### Statistics.

We used GraphPad Prism software v8.0.2 for statistical analysis. Data are presented as mean ± SEM. Experiments using mice were performed at least 2 times and with a group size from 3 to 11 mice. Comparisons were made using 1-way ANOVA with Bonferroni’s or Kruskal-Wallis post hoc test, 2-tailed Student’s *t* test, or paired 2-tailed Student’s *t* test, depending on the set of data. *P* values of less than 0.05 were considered significant.

### Study approval.

Human blood samples were obtained from 34 patients with hemophilia A and 17 healthy individuals (approved by the ethical committee of the medical faculty of the University of Bonn: 313/11 and 327/12). Written informed consent was received prior to participation.

All mouse studies were carried out in accordance with the German animal experimentation law and proven by the relevant local authorities (LANUV, North Rhine-Westphalia).

## Author contributions

JBG and MM share first authorship. JBG wrote the original draft, had the initial conception of the project, and obtained funding for this project; therefore, JBG was assigned first authorship. JBG and CK designed the study. JBG, MM, PAG, and CK wrote the manuscript. FAS, PAG, and BMR provided valuable feedback and corrected the manuscript. JBG, MM, BJM, AP, AM, and VK conducted the experiments. CF assisted with mouse model studies. JBG, JO, and TA designed the human study and provided blood samples of patients. All authors analyzed and interpreted the data. CK and JO supervised all research and are joint senior authors.

## Supplementary Material

Supplemental data

## Version 1 09/15/2022

In-Press Preview

## Version 2 11/15/2022

Electronic publication
