# Supplementary material for: Immune tolerance against infused FVIII in hemophilia A is mediated by PD-L1+ Tregs
Source: J Clin Invest. 2022 Nov 15;132(22):e159925. doi: 10.1172/JCI159925 (PMC9663153; doi:10.1172/JCI159925)
Supplement: Supplemental data [file jci-132-159925-s088.pdf]

Supplemental material

Supplemental Figures

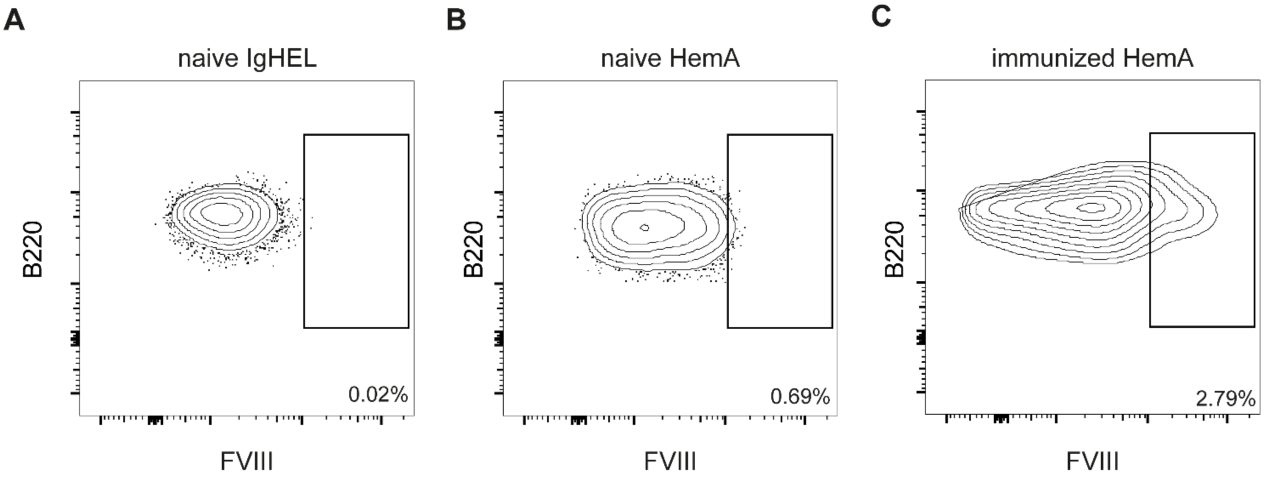

Supplemental Figure 1. FVIII staining specificity. Representative blots of FVIII-specific B cell staining performed on splenic cell suspensions from (A) naïve IgHEL mice (n=3) that express only HEL-specific B cell receptor, (B) HemA mice without (n=3) and (C) Hem A mice injected with 2 UI FVIII per mouse once per week (n=3) are shown.

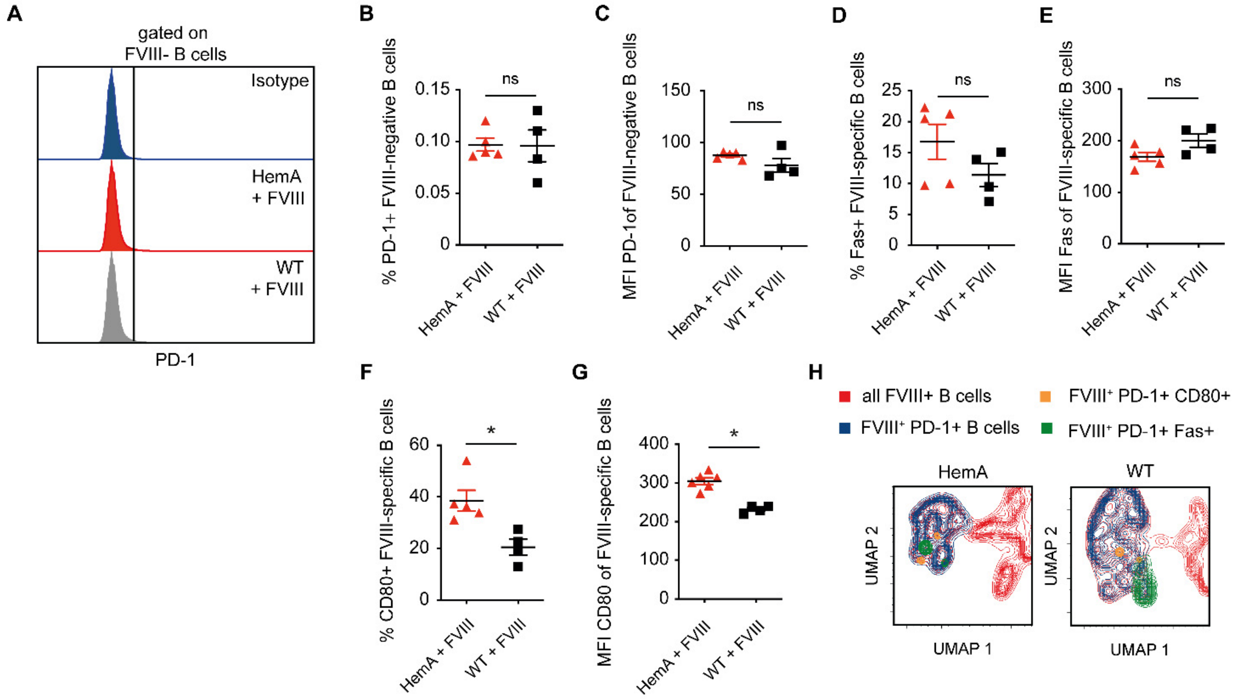

Supplemental Figure 2. PD-1 controls anti-FVIII antibody responses in FVIII-competent mice. Experimental setup for A-H: Two UI/mouse of human rFVIII were intravenously injected into HemA (red, n=5) and WT (black, n=4) in weekly intervals. One day after the last injection splenocytes were further analyzed by flow cytometry. (A) Representative histograms of the PD-1 expression of FVIII-negative B cells. (B) Quantification of the percentage of PD-1 expressing FVIII-negative B cells. (C) Molecular PD-1 expression on FVIII-negative B cells determined by flow cytometry. (D) Percentage of FVIII-specific B cells expressing Fas analyzed at day 22 *ex vivo*. (E) MFI of Fas expressing FVIII-specific B cells. (F) Quantification of the percentage of CD80 expressing FVIII-specific B cells. (G) Molecular CD80 expression on FVIII-specific B cells. (H) Representative UMAPS of FVIII-specific B cells are shown to demonstrate PD-1 single expression or co-expression with CD80 or Fas. P values were calculated using a Students T test. ns…not significant; *P <0.05.

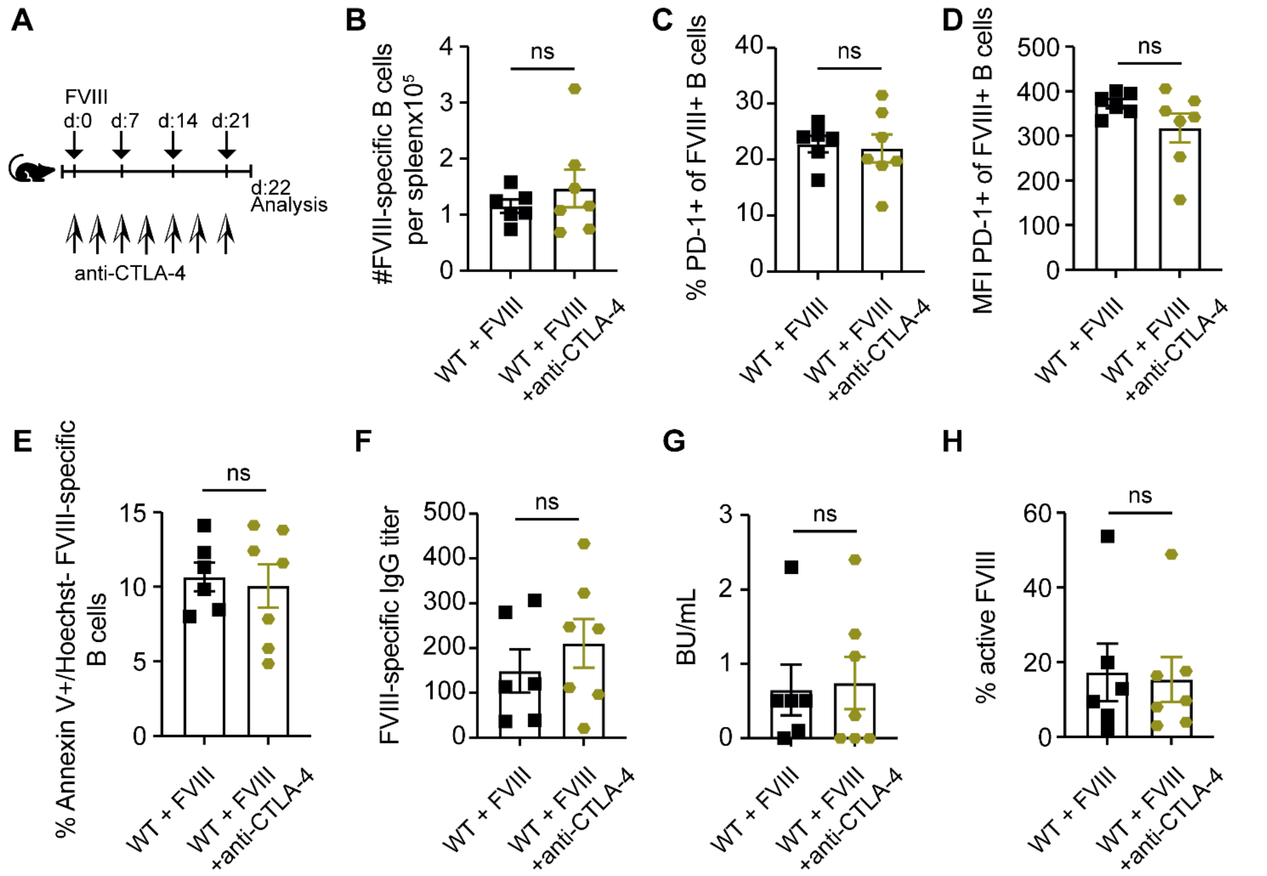

Supplemental Figure 3. CTLA-4 does not regulate inhibitor formation against FVIII. (A) Experimental scheme for B-H: WT mice were injected once a week with 2UI of rFVIII (black, n=6). One group of WT mice additionally received an anti-CTLA-4 inhibitory antibody that was applied twice a week (green, n=7) intraperitoneally. (B) Quantification of the number of FVIII-specific B cells in the spleen of WT mice after treatment with or without anti-CTLA-4 by flow cytometry. (C) Percentage of PD-1 expressing FVIII-specific B cells and (D) MFI of PD-1 on FVIII-specific B cells at day 22. (E) Quantification of the percentage of apoptotic FVIII-specific B cells by analyzing the expression of Annexin V and Hoechst. (F, G) ELISA-based quantification of the FVIII-specific IgG antibody titer (F) and Bethesda units per ml (G) in the serum as well as the percentage of residual active FVIII (H) in the plasma with a COATEST assay in WT and WT mice treated with an anti-CTLA-4 blocking antibody. P values were calculated using a Students T test. ns…not significant P>0,05.

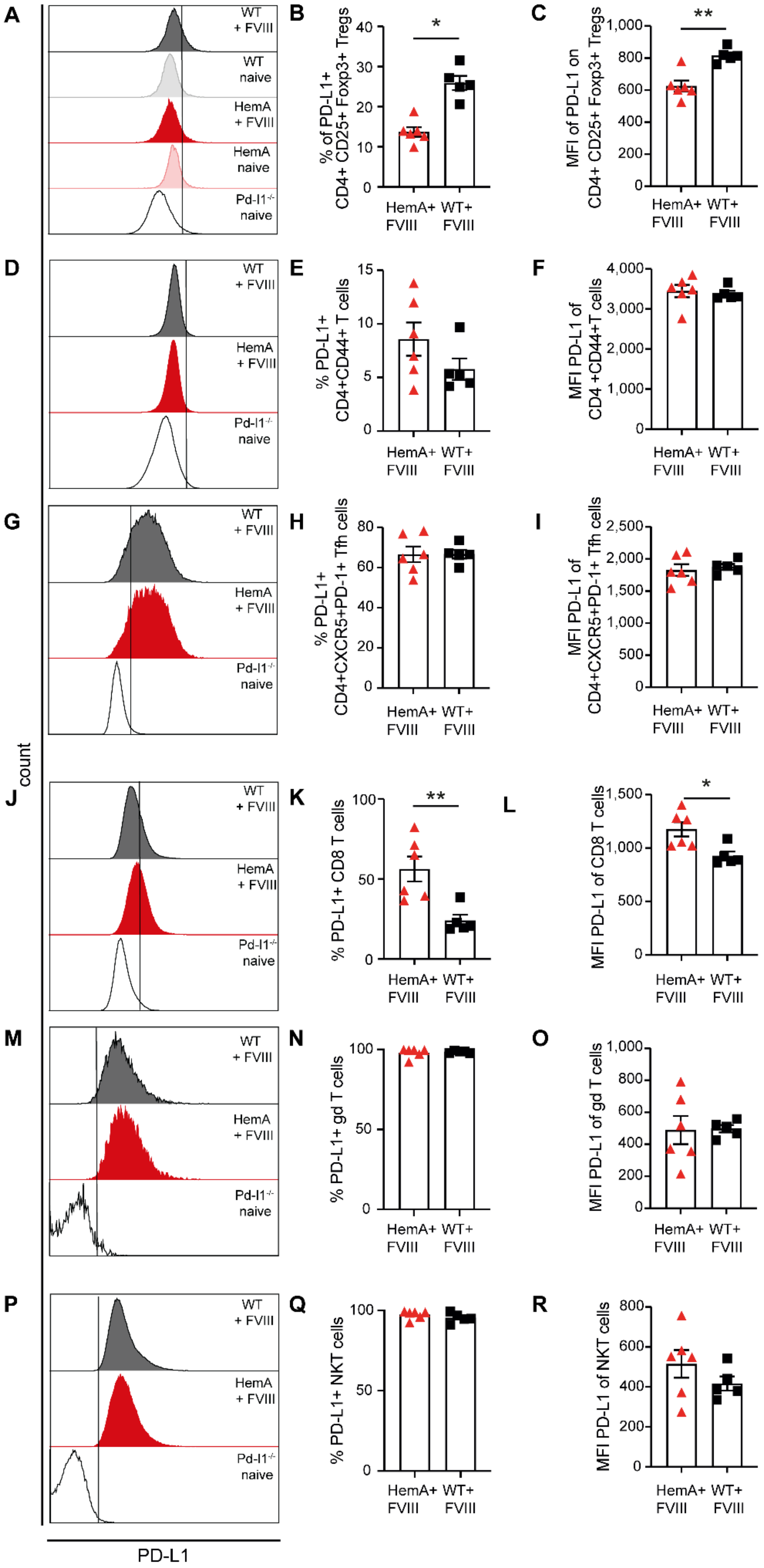
A
WT + FVIII
WT naive
HemA + FVIII
HemA naive
Pd-l1-/- naive
B
% of PD-L1+ CD4+ CD25+ Foxp3+ Tregs
*
HemA+ FVIII
WT+ FVIII
C
MFI of PD-L1 on CD4+ CD25+ Foxp3+ Tregs
**
D
E
% PD-L1+ CD4+CD44+ T cells
F
MFI PD-L1 of CD4 +CD44+T cells
G
H
% PD-L1+ CD4+CXCR5+PD-1+ Tfh cells
I
MFI PD-L1 of CD4+CXCR5+PD-1+ Tfh cells
J
K
% PD-L1+ CD8 T cells
L
MFI PD-L1 of CD8 T cells
M
N
% PD-L1+ gd T cells
O
MFI PD-L1 of gd T cells
P
Q
% PD-L1+ NKT cells
R
MFI PD-L1 of NKT cells
count
PD-L1

Supplemental Figure 4. Enhanced PD-L1 expression on Tregs in WT mice. (A-S) Two UI/mouse of human rFVIII were intravenously injected into HemA (red, n=6) and WT (black, n=5) in weekly intervals. One day after the last injection splenocytes were further analyzed by flow cytometry. Within each histogram, HemA mice are displayed in red and WT mice in grey in comparison to the Pd-l1 knockout control (black line). (A) Representative histograms of the PD-L1 expression on CD4^{+}Foxp3^{+} T cells of immunized mice and naïve HemA mice (light red) or WT control (grey). (B) Percentage of PD-L1 expression and (C) the amount of PD-L1 per cell on regulatory T cells gated as CD4^{+}Foxp3^{+} T cells. (D) Representative histograms of the PD-L1 expression on activated CD4^{+}CD44^{+} T cells. (E) Percentage of PD-L1 expression and (F) the amount of PD-L1 per cell on activated T cells gated as CD4^{+}CD44^{+} T cells. (G) Representative histograms of the PD-L1 expression on follicular T helper cells. (H) Percentage of PD-L1 expression and (I) the amount of PD-L1 per cell on follicular T helper cells gated as CD4^{+}CXCR5^{+}PD-1^{+} T cells. (J) Representative histograms of the PD-L1 expression on CD8 T cells. (K) Percentage of PD-L1 expression and (L) the amount of PD-L1 per cell on CD8 T cells gated as CD8+ T cells. (M) Representative histograms of the PD-L1 expression on gd T cells. (N) Percentage of PD-L1 expression and (O) the amount of PD-L1 per cell on gd T cells gated as CD45^{+}CD3e^{+}gdTCR^{+} T cells. (P) Representative histograms of the PD-L1 expression on NKT cells. (Q) Percentage of PD-L1 expression and (R) the amount of PD-L1 per cell on NKT cells gated as CD45^{+}CD3e^{+}NK1.1^{+} T cells. P values were calculated using a Students T test. *$P < 0.05$; **$P < 0.01$.

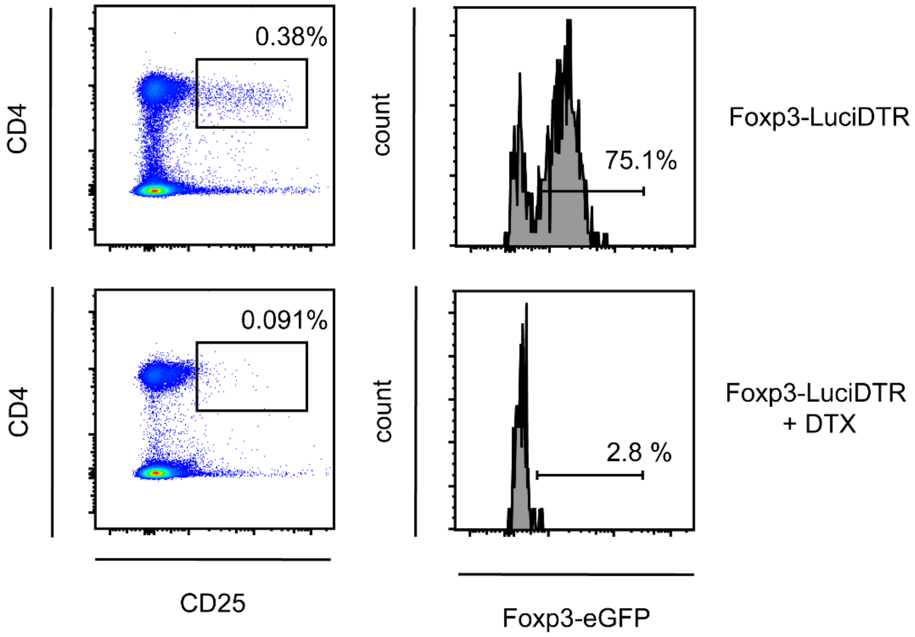

Supplemental Figure 5. Treg depletion efficiency. In Foxp3-LuciDTR mice Foxp3^{+} (n=5) Tregs were depleted by injecting 15 ng/g mouse DTX intraperitoneally at day -1 and 0 of the experiment. Depletion efficiency was controlled in blood samples of DTX treated and untreated mice (n=3) by flow cytometry. Representative Dot blots and histograms are shown. Up to 97% of all eGFP (Foxp3)$^{+}$ Tregs were depleted.

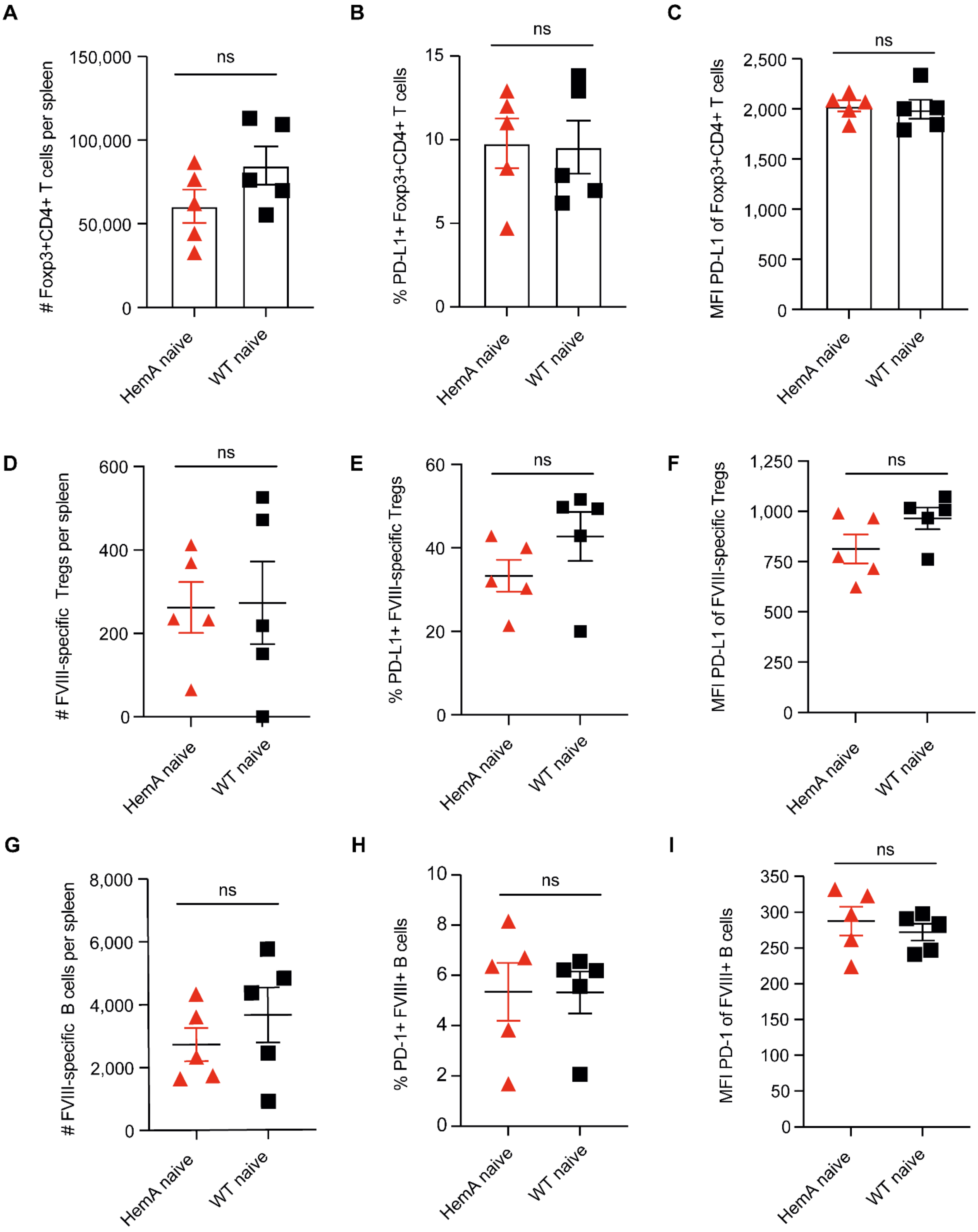
A
Foxp3+CD4+ T cells per spleen
150,000
100,000
50,000
0
ns
HemA naive
WT naive
B
% PD-L1+ Foxp3+CD4+ T cells
15
10
5
0
ns
HemA naive
WT naive
C
MFI PD-L1 of Foxp3+CD4+ T cells
2,500
2,000
1,500
1,000
500
0
ns
HemA naive
WT naive
D
FVIII-specific Tregs per spleen
600
400
200
0
ns
HemA naive
WT naive
E
% PD-L1+ FVIII-specific Tregs
60
40
20
0
ns
HemA naive
WT naive
F
MFI PD-L1 of FVIII-specific Tregs
1,250
1,000
750
500
250
0
ns
HemA naive
WT naive
G
FVIII-specific B cells per spleen
8,000
6,000
4,000
2,000
0
ns
HemA naive
WT naive
H
% PD-1+ FVIII+ B cells
10
8
6
4
2
0
ns
HemA naive
WT naive
I
MFI PD-1 of FVIII+ B cells
350
300
250
200
150
100
50
0
ns
HemA naive
WT naive

Supplemental Figure 6. No differential PD-L1 or PD-1 expression in naïve HemA and WT mice. Splenocytes of naïve HemA (n=5) and WT mice (n=5) were analyzed by flow cytometry. (A) Determination of the number of Tregs gated as $CD4^{+}Foxp3^{+}$, as well as their percentage (B) and their molecular expression of PD-L1 per cell (C). (D) Number of FVIII-specific Tregs gated as single living $B220^{-}$ $CD4^{+}$ $CD25^{+}$ $CD127^{-}$ T cells. (E) Determination of the proportion of PD-L1^{+} FVIII-specific Tregs. (F) PD-L1 expression per cell quantified as gMFI on FVIII-specific Tregs. (G) number of FVIII-specific B cells analyzed by flow cytometry. (H) PD-1 expression shown as % and (I) MFI of PD-1 on FVIII-specific B cells. P values were calculated using a Students T test. ns...not significant P >0.05.

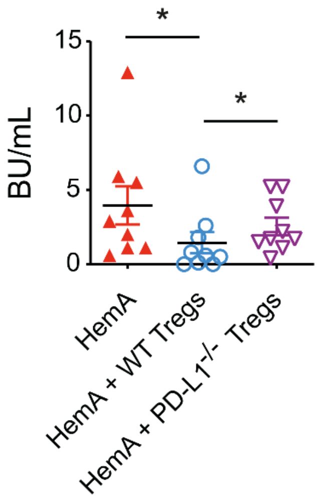

Supplemental Figure 7. PD-L1 competent Tregs reduce inhibitor titers in HemA mice. Tregs were isolated either from WT or PD-L1$^{-/-}$ mice and 1x10^{6} cells were transferred into HemA mice by intravenous injections. Starting from the next day, HemA (red, n=9) and HemA mice that received Tregs from WT (blue, n=9) or PD-L1$^{-/-}$ (purple, n=9) mice were intravenously injected with 2 UI/mouse of rFVIII in weekly intervals. One day after the last injection blood serum was harvested. Bethesda units per ml were measured by ELISA in serum of the indicated mice. P values were calculated using one-way ANOVA and Bonferroni posttest. *P <0.05.

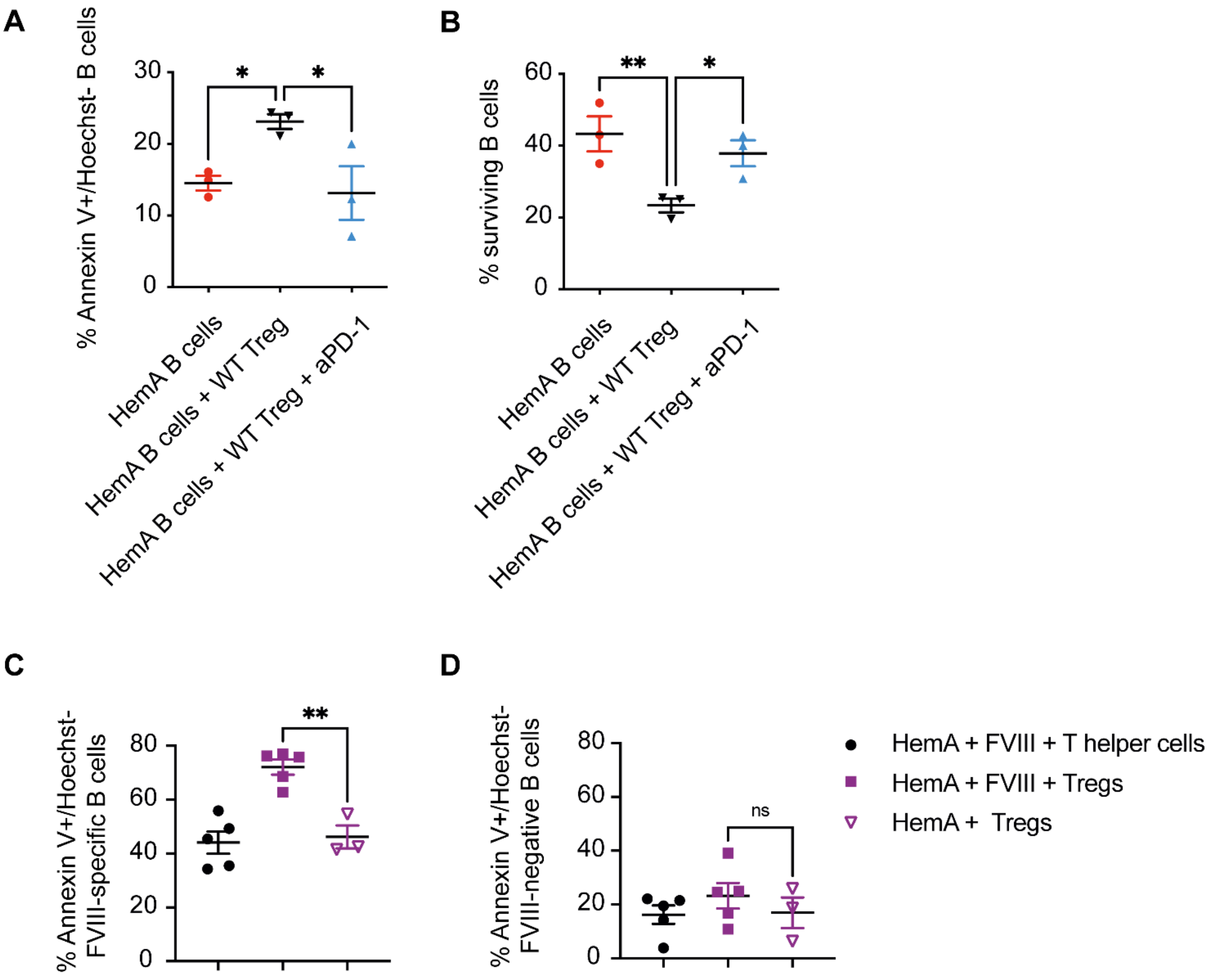

Supplemental Figure 8. Treg-mediated suppression of antibody-producing B cells is PD-1 dependent. HemA mice (n=3) were immunized 3 times with 2UI of FVIII in a weekly interval. Afterwards, plasma cells were isolated from splenic cell suspensions and $3x10^5$ B cells were cocultured with $5x10^5$ Tregs isolated from naïve WT mice for 16 hours with or without a PD-1 blocking antibody (RPM1-14; 40 µg/ml). (A) Percentage of early apoptotic B cells after co-culture. (B) overall percentage of surviving B cells after 16 hours of co-culture. (C and D) $0,5x10^6$ naïve splenocytes from HemA mice (n=5) were co-cultured with either $0,5x10^6$ wildtype Tregs or T helper cells for 16 hours with or without FVIII protein. Quantification of the proportion of early apoptotic FVIII-specific B cells (C) or FVIII-unspecific B cells (D). P values were calculated using one-way ANOVA and Bonferroni posttest. ns...not significant P >0.05, *P <0.05, **P < 0,001.

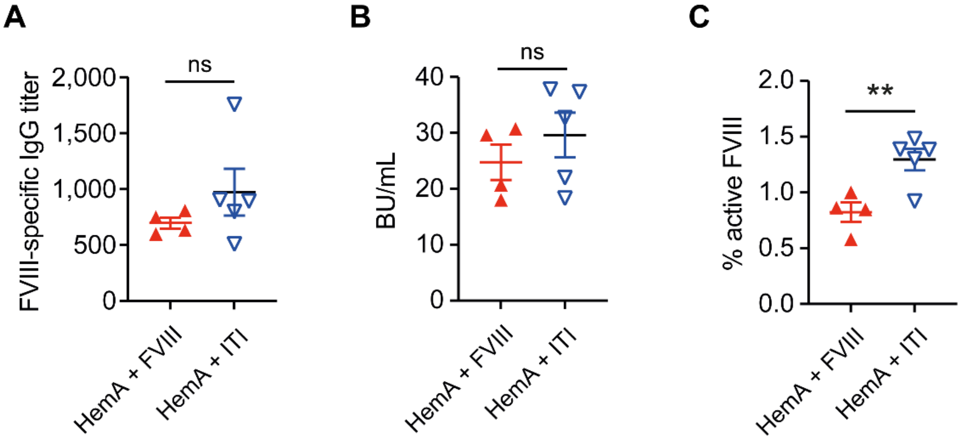

Supplemental Figure 9. ITI-like treatment increases active FVIII levels but does not diminish inhibitors in HemA mice. Experimental setup for A-C: 2 UI/mouse of human rFVIII were intravenously injected into HemA (red, n=4) mice in weekly intervals for three weeks, and twice a week for the high dose FVIII regime (blue, n=5). Compared to the therapeutic FVIII treatment (once a week), the high dose FVIII application regime (twice a week) is used to induce tolerance (short ITI) in HemA mice. One day after the last injection blood serum and plasma were harvested and further analyzed by ELISA or COATEST assay, respectively. (A) Quantification of the FVIII-specific IgG antibody titer and (B) Bethesda units per ml in the serum. (C) The percentage of residual active FVIII was determined in the plasma of HemA mice treated with FVIII once or twice a week. P values were calculated using a Students T test. ns…not significant; **$P < 0.01$.

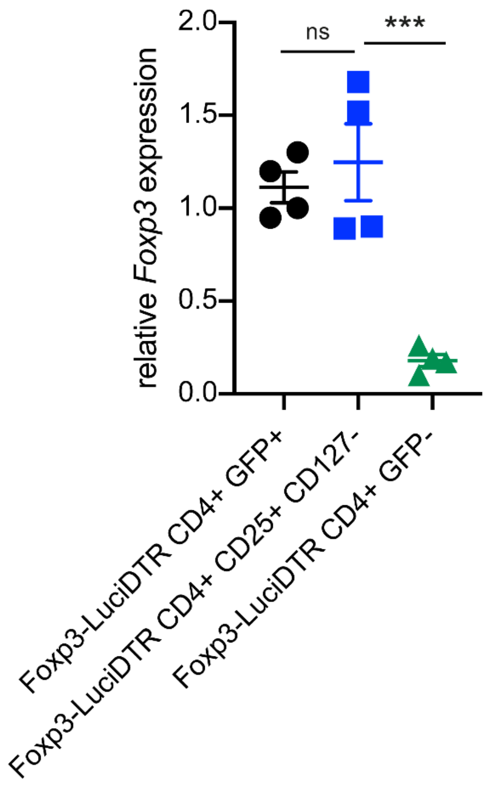

Supplemental Figure 10. Foxp3 expression of sorted CD25^{+}CD127^{-} CD4^{+} T cells. Regulatory T cells of Foxp3-LuciDTR mice (n=4) were sorted either as CD4^{+}eGFP^{+}(Foxp3^{+}) or as CD4^{+}CD25^{+}CD127^{-} T cells. As a control CD4 T helper cells were sorted as CD4^{+} eGFP^{-}(Foxp3^{-}) T cells. RNA isolation and a subsequent qRT-PCR was performed to determine the expression of *Foxp3* in these cells. Expression is correlated to one mean of Tregs sorted as CD4+eGFP+ T cells. P values were calculated using one-way ANOVA and Bonferroni posttest. ns…not significant P>0,05; ***P < 0.001.

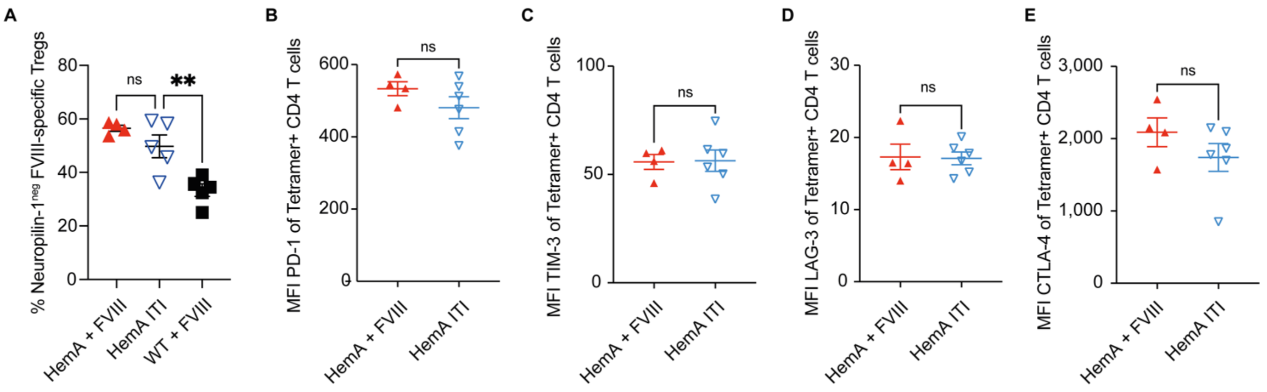

Supplemental Figure 11. ITI-induced Tregs are mainly peripherally derived Tregs. (A) 2 UI/mouse of human rFVIII were intravenously injected into HemA mice (red, n=4) or WT mice (black, n=5) in weekly intervals for 3 weeks, and twice a week for the high dose FVIII regime (blue, n=5). At day 22, splenic suspensions were analyzed by flow cytometry to discriminate the proportion of peripherally-derived (Neuropilin-1 negative) FVIII-specific Tregs. FVIII-specific Tregs were identified as living, single B220-CD4+CD25+CD127- T cells. Error bars represent SEM. P values were calculated using one-way ANOVA and Bonferroni posttest. ns…not significant, **P < 0,001. (B-E) 2 UI/mouse of human rFVIII were intravenously injected into HemA mice (red, n=4) in weekly intervals for 3 weeks, and twice a week for the high dose FVIII regime (blue, n=6). At day 22, splenic suspensions were analyzed by flow cytometry to determine the expression of the exhaustion markers PD-1 (B), TIM-3 (C), LAG-3 (D) and CTLA-4 (E), per cell on living, single FVIII-specific (tetramer+) CD4 T cells. P values were calculated using a Students T test. ns…not significant P>0,05.

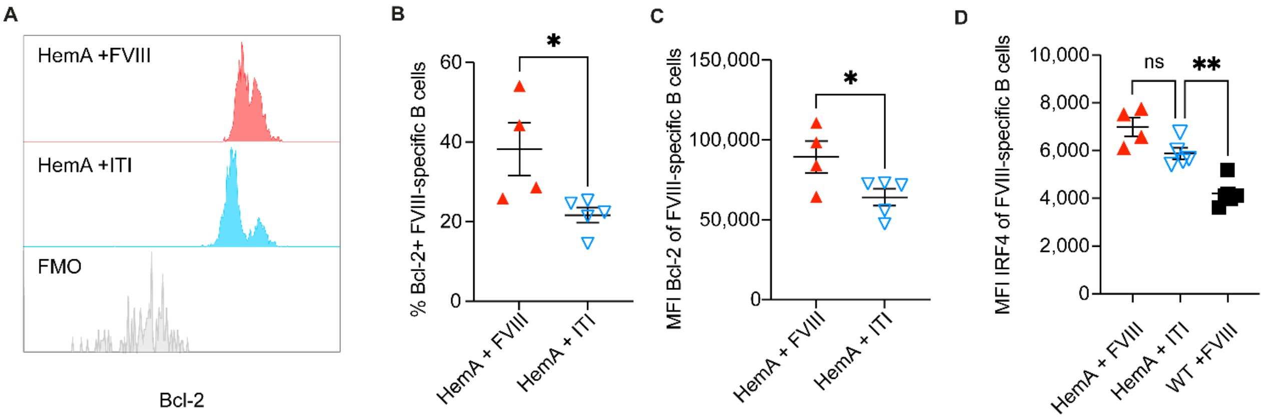

Supplemental Figure 12. Decreased Bcl-2 expression in FVIII-specific B cells of ITI-treated mice. (A-C) Two UI/mouse of rFVIII were intravenously injected into HemA (red, n=4) mice in weekly intervals. Immune tolerance induction in HemA mice (blue n=5) was achieved by injecting of rFVIII twice a week. (A) Representative histograms of Bcl-2 expression in FVIII-specific B cells. (B) Quantification of the proportion of Bcl-2 expressing FVIII-positive B cells. (C) Mean Bcl-2 expression per cell in FVIII-positive B cells. P values were calculated using a Students T test. *P < 0.05 (D) Two UI/mouse of rFVIII were intravenously injected into HemA (red, n=4) or WT (black, n=5) mice in weekly intervals. Tolerized HemA mice (blue n=5) were injected twice a week with rFVIII. Mean expression of IRF4 per cell in FVIII-positive B cells is displayed. P values were calculated using one-way ANOVA and Bonferroni posttest. **P < 0,001

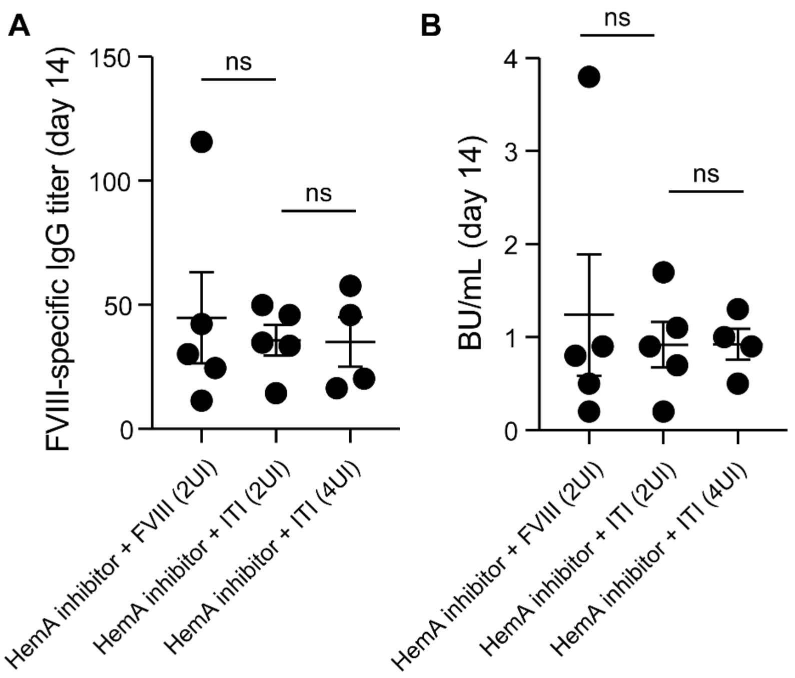

Supplemental Figure 13. Inhibitor formation in HemA mice. Two UI/mouse of human rFVIII were intravenously injected into HemA mice (n=14) in weekly intervals. HemA mice were bled 14 days after initial injection to measure IgG antibodies (A) and inhibitor production (B) in the serum of the mice via ELISA. Based on these results the mice were equally distributed amongst all three group to obtain a comparable pre-treatment titer as depicted in A and B. P values were calculated using one-way ANOVA and Bonferroni posttest. ns…not significant P>0,05.

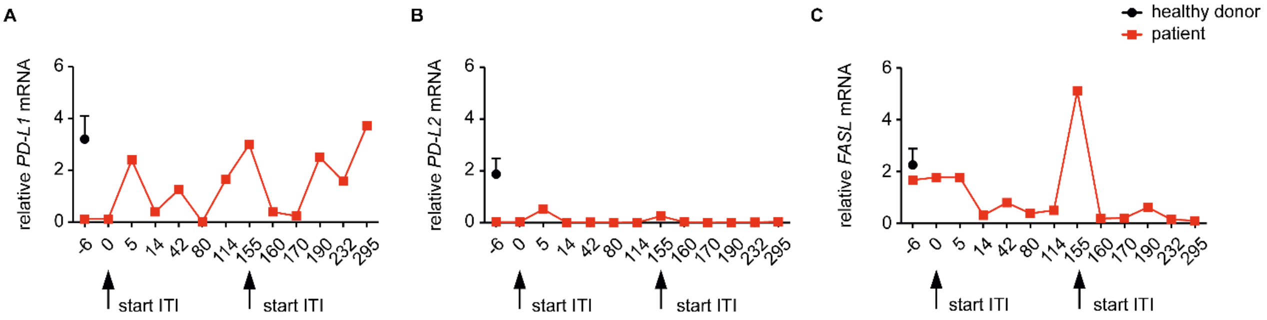

Supplemental Figure 14. Inhibitory ligand expression on FVIII-specific B cells in humans. (A-C) mRNA was extracted from sorted FVIII-specific B cells of the patient on ITI shown in Figure 6B (red) or healthy controls (black) and analyzed by RT-PCR at different time points during ITI. Relative mRNA expression of *PD-L1* (A), *PD-L2* (B), and *FASL* (C) in FVIII-specific B cells. Expression is correlated to one healthy individual. Data are presented as mean +/- SEM.

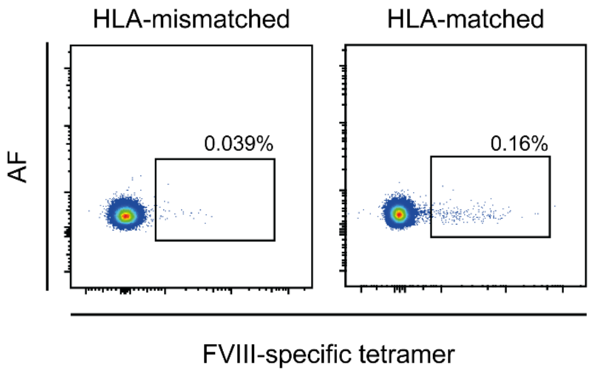

Supplemental Figure 15. FVIII-specific tetramer staining of CD4 T cells in humans. Blood was collected from HLA-matched (HLA15.01, n=3) or -mismatched hemophilia A patients (n=3). The specificity of the tetramer staining was verified by flow cytometry. The cells were gated on living single cells and the binding capacity is displayed on CD4$^+$ T cells. Representative dot plots are shown.
